# Protein Immobilization on Bacterial Cellulose for Biomedical Application

**DOI:** 10.3390/polym16172468

**Published:** 2024-08-30

**Authors:** Anastasia N. Shishparenok, Vitalina V. Furman, Natalia V. Dobryakova, Dmitry D. Zhdanov

**Affiliations:** 1 Institute of Biomedical Chemistry, 10/8 Pogodinskaya St., 119121 Moscow, Russia; a.shishparyonok@ibmc.msk.ru (A.N.S.); natdobryak@gmail.com (N.V.D.); 2 The Center for Chemical Engineering, ITMO University, 197101 Saint Petersburg, Russia; furmanvivi@gmail.com; 3 Department of Biochemistry, People’s Friendship University of Russia Named after Patrice Lumumba (RUDN University), Miklukho-Maklaya St. 6, 117198 Moscow, Russia

**Keywords:** bacterial cellulose, protein immobilization, bacterial cellulose modification, biomedicine

## Abstract

New carriers for protein immobilization are objects of interest in various fields of biomedicine. Immobilization is a technique used to stabilize and provide physical support for biological micro- and macromolecules and whole cells. Special efforts have been made to develop new materials for protein immobilization that are non-toxic to both the body and the environment, inexpensive, readily available, and easy to modify. Currently, biodegradable and non-toxic polymers, including cellulose, are widely used for protein immobilization. Bacterial cellulose (BC) is a natural polymer with excellent biocompatibility, purity, high porosity, high water uptake capacity, non-immunogenicity, and ease of production and modification. BC is composed of glucose units and does not contain lignin or hemicellulose, which is an advantage allowing the avoidance of the chemical purification step before use. Recently, BC–protein composites have been developed as wound dressings, tissue engineering scaffolds, three-dimensional (3D) cell culture systems, drug delivery systems, and enzyme immobilization matrices. Proteins or peptides are often added to polymeric scaffolds to improve their biocompatibility and biological, physical–chemical, and mechanical properties. To broaden BC applications, various ex situ and in situ modifications of native BC are used to improve its properties for a specific application. In vivo studies showed that several BC–protein composites exhibited excellent biocompatibility, demonstrated prolonged treatment time, and increased the survival of animals. Today, there are several patents and commercial BC-based composites for wounds and vascular grafts. Therefore, further research on BC–protein composites has great prospects. This review focuses on the major advances in protein immobilization on BC for biomedical applications.

## 1. Introduction

Protein immobilization is a biotechnological technique in which a protein is fixed in a suitable matrix to limit mobility, increase stability, and, in the case of an enzyme protein, allow reuse while retaining the immobilized protein activity [1]. The immobilized proteins and enzymes have been used in biomedicine, including the detection and treatment of many diseases. The immobilization process allows protein functionality to be optimized for specific tasks. For example, immobilized antibodies, receptors, or enzymes are used in biosensors and ELISA to detect various bioactive substances in disease diagnosis. Immobilized enzymes are also used in bioreactors to remove waste metabolites and correct congenital metabolic deficiencies. Today, artificial cells are being developed and controlled-release drug delivery systems based on the release of encapsulated enzymes or proteins are being created [2,3]. Immobilization of the protein improves its stability by preventing environmentally induced structural denaturation, allowing it to remain active under various conditions [4,5]. The incorporation of proteins into polymer biomaterials enables the acquisition of pro-adhesive properties, a modification of the biomaterial’s hydrophilicity, the introduction of supplementary functional groups that facilitate cellular activity, an alteration of the surface stiffness, and a modification of the biomaterial’s degradation rate [6].

The matrix for protein immobilization should be inert, stable, accessible, resistant to mechanical stress, and biocompatible without disrupting the protein structure. Among the matrixes, natural polymers as carriers have attracted considerable attention [7]. Polysaccharides, cellulose, chitosan, alginate, and their derivatives are widely used in protein immobilization [8]. Recently, cellulose materials have been studied and used due to their good renewability, availability, biodegradability, and biocompatibility [9]. Cellulose is a robust, dual hydrophilic/hydrophobic, non-toxic, and chemically inert material under physiological conditions [10]. Cellulose is a versatile material that can be used as a reinforcement material or as a matrix, depending on the specific requirements of the task at hand. Its well-organized fibrous network structure allows it to enclose nanoparticles, acting as a matrix. Similarly, cellulose nanofibers can be used to reinforce other materials, cells, and tissues [11]. BC has similar chemistry and superior physical properties to plant cellulose, making it an ideal choice for manufacturing a range of composite materials for diverse applications [12]. The composite BC with protein can utilize the dual properties of both materials [13]. In addition, several BC-based composites were created that showed high protein immobilization efficiency compared to other materials [14,15,16].

BC is produced by gram-positive bacteria such as Sarcina and gram-negative bacteria strains the Acetobacter, Rhizobium, Agrobacterium, Aerobacter, Achromobacter, Azotobacter, Salmonella, Escherichia [17,18]. Moreover, BC can be synthesized in a cell-free system [19]. BC is a biopolymer consisting of linear chains of covalently linked glucose residues between carbon 1 and 4—β (1,4)-bound d-glucopyranosyl [20]. BC does not contain lignin and hemicellulose, rendering it more pure than plant cellulose [21]. BC has high porosity, water-holding capacity biocompatibility, low toxicity, and non-immunogenicity and can be readily and safely sterilized [22]. Moreover, BC has enhanced mechanical strength, biodegradability, and high crystallinity [14] and increased the surface-to-volume ratio [23,24].

BC composites are prepared to adapt their characteristics for a specific application [25] and overcome the disadvantages of native BC. By in situ modification, different structures can be formed during fermentation to produce materials for various applications. In addition, BC can be modified ex situ by adding a functional compound or polymer coating [26]. Previously has been demonstrated that the combination of nanoparticles (Ag, ZnO, etc.) and polymers (e.g., polyaniline, chitosan, polyethylene glycol) can be used to create BC composites with bactericidal and conductive properties [12], enhance antimicrobial, antiviral and anticancer activities [27]. BC-based nanocomposites were used for phage immobilization to detect live *S. aureus* [28], incorporation of probiotic bacteria *Lactobacillus acidophilus* 016 [29], and yeast immobilization [30]. BC is utilized in the development of drug delivery systems, scaffolds, tissue and organ regeneration, and wound healing [31].

The latest drug delivery research has further demonstrated various advantages of BC, including its promising effects on controlling drug release, biocompatibility, low immunogenicity, and ease of production and handling [32]. BC films have been successfully used for the immobilization of antibiotics (amoxicillin [33], tetracycline [34], levofloxacin [35], ciprofloxacin [36], gentamycin [37]), anesthetics (lidocaine [38]), anticancer drugs (doxorubicin [39], curcumin [40], paclitaxel [41], immune checkpoint blocking antibodies [32]). The porous structure and increased surface area of BC make it useful for the immobilization of enzymes and bioactive compounds [42]. Various types of BC and activation methods have been investigated to immobilize glucoamylase [43,44], glutamate decarboxylase [45], and laccase [14].

The commercialization of BC has increased markedly recently, driven by its growing applications in a range of fields. The global BC market was valued at approximately USD 426.7 million in 2022 [46] and is projected to reach approximately USD 777 million by 2027 [47]. Among the major trends in the BC market today, the main interest is in the creation of BC-based composites for wound care products, dental treatment, organ regeneration, and BC for drug delivery [46]. Soon, BC can potentially be widely applied in bioprinting techniques [48]. Furthermore, as regulatory agencies promote the use of xeno-free biomaterials in human care and medicine delivery, BC has gained popularity due to its animal- and human-free origin [49]. Currently, an increasing number of studies [50] have been dedicated to the utilization of BC in the development of protein composites for wound healing, tissue engineering, three-dimensional (3D) cell culture systems, drug delivery systems, and enzyme immobilization matrices. This review concentrates on the employment of BC for protein immobilization in biomedical applications, the diverse forms of protein immobilization and the modifications of BC for protein immobilization, and the advantages and disadvantages of employing BC as a carrier.

## 2. Structure of Bacterial Cellulose

BC is a linear glucose biopolymer produced primarily by the bacterium *Gluconacetobacter xylinus* (*G. xylinus*) in both synthetic and non-synthetic environments via oxidative fermentation. This non-photosynthetic organism can obtain sugars, glycerol, glucose, and various other organic molecules before converting them to pure cellulose [51,52]. Bacteria strains typically produce and use BC as a protective envelope against harsh environmental conditions or as a component of their cell walls. The bacteria release nanofibrils that self-assemble into larger fibers that interconnect to form a BC matrix of increasing volume and density. The BC matrix forms a visible hydrogel called a pellicle at the air–liquid interface of the working culture [53].

**Figure 1:**
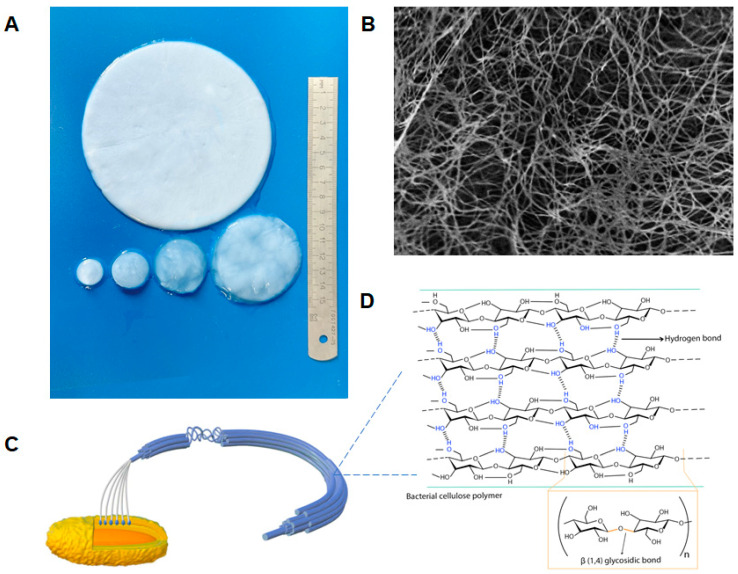
Schematic of bacterial cellulose structure: (**A**) Photograph of purified BC films. (**B**) SEM image of films grown for 72 h. Magnification 10,000×; (**C**) Schematic of the structure of BC microfibrils. BC are arranged in parallel chains (10–15) via hydrogen bonds to form subfibrils, then aggregated into microfibrils, and finally into microfibril bundles that form a ribbon composed of about 1000 individual glucan chains [56]; (**D**) Chemical structure of the BC polymer and inter- and intra-hydrogen bonding of BC. Intramolecular hydrogen bonds form in the C3–OH group and oxygen on the pyranose ring.

BC is composed of β-(1 → 4) glucan chains with fiber widths of 25–100 nm and lengths of several microns. It is predominantly twisted to the left. BC is the “gold standard” for nanocellulose because one of its dimensions is on the nanometer scale, and it is released in a controlled environment by bacteria. The monomer unit has a cyclic structure with reactive primary and secondary hydroxyl groups (Figure 1). The β-D-glucopyranose ring and all -OH groups are free and play an important role in intermolecular hydrogen bonding between adjacent chains [20]. Each monomer unit is rotated 180° relative to its neighbor. These chains first form nanofibrils, then microfibrils, and finally macrofibrils (Figure 1) [54]. The microfibrils exhibit distinctive characteristics, including unidirectional polarity and variable thickness [55].

Although both BC and plant cellulose have structural similarities, their distinct nanofibrous structures give them different physical and chemical properties [57]. BC is classified as a cellulose subtype Iα, while plant cellulose is mainly classified as Iβ, which differ in their crystalline structure [54], molecular conformation, and hydrogen bonding, and these differences may influence the physical properties of the cellulose [56]. In contrast to plant cellulose, BC comprises a fully crystalline core encased in a less crystalline zone interposed by the amorphous form of cellulose, along with fibers arranged in a 3D lattice. Due to the robust intermolecular interactions and the presence of hydroxyl groups, the fibers exhibit a proclivity for self-assembly. These fibers give rise to a network structure interconnected by intramolecular hydrogen bonds, resulting in the formation of sheets with a high surface area and porosity [20].

BC exhibits ultrafine fibers (width 50–80 nm and thickness 3–8 nm) [14], and BC is thinner than plant cellulose [23,24]. Furthermore, in contrast to plant cellulose, which typically consists of fibers with 10,000–15,000 degrees of polymerization, BC fibers consist of the pure form of the polymer with a degree of polymerization of 2000–8000. Such structural characteristics render microbial cellulose with improved mechanical properties, such as strength and toughness, compared to plant cellulose with a similar chemical structure [53]. Different morphologies of BC have a direct effect on its mechanical properties and cell attachment to the material. Tang et al. [58] produced BC mats with different aperture sizes and porosities when the fermentation conditions and post-treatment interventions were changed. Backdahl et al. [59] invented a novel approach to introduce microporosity in BC tubes, which are intended to be used as scaffolds for tissue-engineered blood vessels [51]. BC, like plant cellulose, contains reactive hydroxyl groups that can be modified [25]. The increased surface area-to-volume ratio allows for greater interaction with the components of BC-derived composites, making them ideal for medical applications.

## 3. Biomedical Application of Bacterial Cellulose

The use of BC for biomedical applications has attracted considerable interest over the past decade [60]. BC is a promising biomaterial due to its physical and chemical properties [24,25,61]. Various surface functionalizations by biosynthetic or chemical modification can enhance the functionality of BC and broaden their potential applications [62].

**Figure 2:**
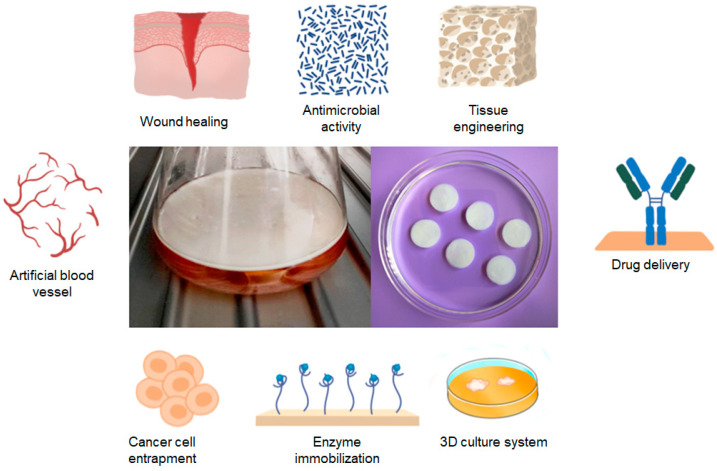
The primary areas of application for BC in the biomedical industry.

Both native and modified BC have been used in various applications, including tissue engineering, the fabrication of artificial organs, and the development of scaffolds and drug delivery systems (Figure 2) [63]. Previously, the most diversified application of BC in biomedicine has been the creation of drug delivery systems. A significant number of published research studies have attempted to develop an effective BC-based system for delivering specific drugs to the wound or diseased tissue [64]. The majority of BC carrier research focuses on loading cellulose (often a dressing) with small molecules, including analgesics and anti-inflammatory drugs, bacteriostatic agents such as metal ions, antibiotics, or other chemicals [65].

The latest developments in the field of BC focus on new carrier materials for cell culture, cell encapsulation or enzyme immobilization, medical device coating, and the production of BC materials with soft tissue implants and materials for bone regeneration [66]. To achieve this, it is important to immobilize larger molecules such as proteins (e.g., albumin, lysozyme, lipase, or phospholipase) and growth factors in the membrane after changing the porosity of the cellulose nanostructure. In recent times, BC-based materials have been employed in the field of cancer therapeutics [24,61]. BC-based materials are also suitable for the delivery of proteins and nucleic acids [67,68].

### 3.1. Wound Healing and Antibacterial Wound Dressings

A wound is a break in the continuity of the skin. Microbial infection and inflammation are the most common wound-related conditions [69]. Wound healing requires the coordinated and balanced activity of inflammation and vascularization of connective tissue and epithelial cells. All of these stages require an extracellular matrix (ECM) and various growth factors to support the healing process [70]. The primary proteins of the ECM are collagen, non-collagen fractions, and proteoglycans [71]. These proteins serve as scaffolds and mediate cell attachment, cellular proliferation, differentiation, and migration. Each organ has a unique ECMs [20].

ECM proteins, a plethora of growth factors, and antidiabetic wound-healing agents (insulin) play a pivotal role in the process of tissue regeneration [72]. Proteins and growth factors are used to create nanopreparations with wound healing and regenerative effects to help reduce inflammation, cell proliferation, and remodeling. These formulations release proteins over time, increasing the efficacy and impact on the wound area, promoting targeted drug delivery, improving solubility and biocompatibility, and helping wounds heal without complications [72]. Enzymatic wound treatment with proteases is already used in surgical practice [73]. Proteolytic enzymes (serine proteases, metalloproteinases, cysteine proteases, and aspartate proteases) contribute positively to tissue regeneration processes [74].

Despite the advances in wound healing management, the treatment of the majority of skin lesions remains a significant challenge for the biomedical and pharmaceutical industries [75,76,77]. Alternative approaches, such as the use of hydrogels as wound dressings, can be used when surgery is not feasible due to patient circumstances or when there is insufficient necrotic tissue [78]. Wound healing materials must be able to keep the wound moist, absorb wound fluid, promote new skin growth, let in oxygen and other gases, and fight infection. They must also be safe for the body [79]. In addition, the material must act as a suitable interphase to support the complex interactions that occur during wound healing involving various cells, soluble substances, and ECM components. Therefore, the aforementioned properties of BC make it ideal for this purpose [25,80].

For the first time, BC was used in medicine as a wound dressing to promote tissue regeneration [81]. A moist environment, non-toxicity and non-allergy, promotion of thermal insulation, and ease of gas transfer are the primary requirements for effective wound dressings [82]. The use of constructed BC composite scaffolds can extend cell adhesion, proliferation, and transplantation of scaffold-seeded cells, enhancing their biocompatibility [81]. It has been shown that BC and BC-composites are biocompatible with skin tissues [83]. BC is appropriate for skin care applications because it can relieve discomfort, speed healing, and fit the body well [82]. Water-holding is an important factor in the healing process because dry wounds need more moisture to promote tissue regeneration and prevent necrosis [84,85]. BC has a 30% higher water absorption capacity and a 33% longer drying time than cotton gauze [86]. In terms of maintaining a moist wound environment, reducing discomfort, accelerating tissue re-epithelialization, and minimizing scarring, BC-based wound dressings outperformed conventional wound dressings [31,87]. By retaining liquid, a hydrogel can be used to load liquid medications and physiologically active substances close to the dressing material [88].

In vivo studies have proved that BC wound dressings in porous form outperform compacted form in terms of wound healing performance [13,89]. Long-term clinical trials have shown that BC-based dressings are more cost-effective than standard fiber dressings (surgical pads, tulle grass, and saline-impregnated gas), synthetic foams, and alginate dressings. The high surface area and porosity of BC allow for the integration of additional wound-healing-promoting agents [12,90]. A number of commercial BC-based dressings have been developed, including BioFill^®^, Bioprocess^®^, Gengiflex^®^ (BioFill Produtos Biotecnológicos, Curitiba, Brazil), Xcell^®^ (Xylos Corporation, Langhorne, PA, USA), Dermafill^®^ (Seven Indústria de Produtos Biotecnológicos Ltda, Londrina, Brazil), and Epiprotect^®^ (Royal Wootton Bassett, UK). The efficacy of BC-based dressings has been investigated in clinical trials, with the results demonstrating that the BC membrane can significantly reduce pain and facilitate the healing of a range of wounds [83,91]. The dressings based on BC can vary considerably in composition, from relatively simple films to highly complex constructions. They often incorporate various biomolecules, pharmaceutical agents, and polymers [92].

There are several limitations to the use of BC-based dressings. Due to the slow degradation of BC, several attempts have been made to improve the degradability of BC for wound healing. Several approaches have been used: oxidation with periodate, γ-irradiation, or incorporation of the enzyme cellulase into BC [93]. Compared to more expensive protein-based materials, BC shows limited cell attachment and proliferation, especially in fibroblast cells in the wound. Several modification strategies have been developed to improve the cellular response of fibroblasts to various materials. Techniques include biopolymer/protein adsorption, gas plasma surface modification, and self-assembled monolayers [94].

**Figure 3:**
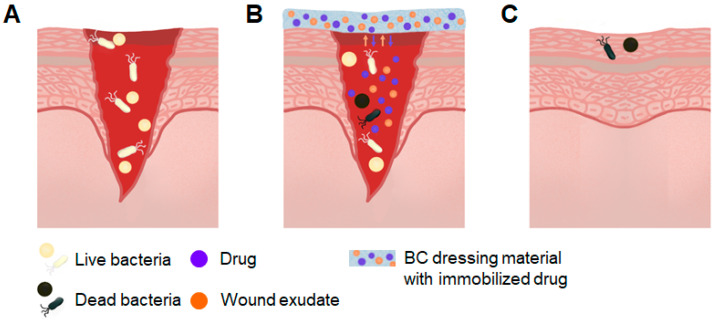
Schematic of wound treatment with antibacterial BC-based composites. (**A**) Infection of open wound; (**B**) wound treatment with BC composite. The BC absorbs wound exudate and is loaded with an antibacterial agent that is released into the wound; (**C**) healed wound.

Regarding passive wound healing, BC can be combined with various compounds, including antimicrobial polymers, photosensitizers, metallic nanoparticles, antibiotics, antimicrobial peptides, and antiseptics to accelerate wound healing. Researchers often use BC doped with conductive chemicals to locate wound healing sites. This allows the circuit to be carried over a large area and stimulates skin cell behavior, resulting in faster healing [81,91]. BC-based composites can help retain and slowly release antibiotics (Figure 3); however, the issue of antibiotic resistance is still being investigated [95]. Antibacterial nanomaterials may replace antibiotics, but nanoparticles have drawbacks such as easy aggregation, an unpredictable tendency to release ions, and potential cytotoxicity that limit their use [96]. Due to the large number of active functional groups, BC and its modified derivatives can be used as a template or immobilization material for antibacterial nanoparticles, which helps to reduce nanoparticle agglomeration and control the release rate. As a result, there is no defect-free BC-based wound dressing material, and the development of multifunctional BC-based composites is an important area of future research [97].

There are a number of techniques that can be employed to modify BC in order to address the shortcomings of the native material while simultaneously optimizing its biocompatibility, water uptake and release, and antibacterial activity [97]. Given that BC lacks intrinsic antimicrobial properties [78], it is modified by incorporating other polymers or inorganic materials. Modification of BC has several goals, including improving mechanical properties, changing some physical parameters such as water-holding capacity, water retention rate, and water vapor transmission rate, or even imparting antimicrobial activity [98]. Various techniques, including microbial fermentation, physical modification, chemical modification, and combination modification, have been used to enhance the biocompatibility and antibacterial activity of BC to ensure better use in wound healing [25,83]. For dental and drug delivery applications, the degradability of BC mediated by oxidation is very important [25], as BC is slow to degrade in the human body [99,100]. Also, composites are created with collagen [101,102,103,104] and sericin [105]. These polymers strengthen the BC structure, improve its mechanical properties, and accelerate wound healing [78,106]. β-keratose [107], ECMs (collagen, elastin, and hyaluronan) and growth factors (B-FGF, H-EGF, and KGF) [70], laccase [108], soybean protein isolate [13], involucrin antibody SY5 [109], papain [110] have been used for the development of BC-based wound dressings. The BC with antibacterial activity was obtained with lysozyme [111,112] and such peptides as nisin [113], ε-poly-l-Lysine [79], and bacteriocins from *Lactobacillus sakei* [114,115].

### 3.2. Tissue Engineering

Tissue engineering involves developing scaffolds and growth factors that influence the regeneration or replacement of damaged tissue [106,116,117]. The scaffold material plays a pivotal role in providing the biological and physical environment essential for tissue growth, including the ECM. This is achieved by facilitating cell adhesion, development, and differentiation [71,118]. The replacement of natural ECMs is becoming an increasingly crucial and promising aspect of tissue engineering, as they facilitate the localization and transportation of cells to specific regions of the body [119].

**Figure 4:**
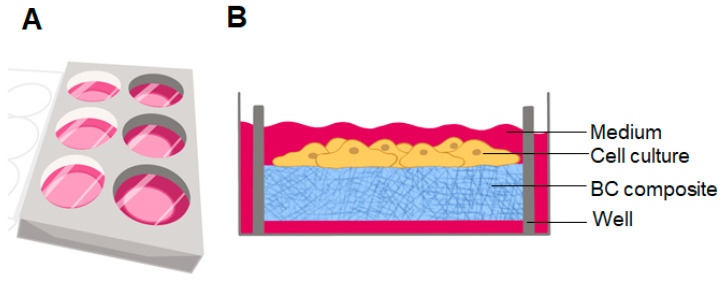
(**A**) Culture plate with BC hydrogel placed in a black well; (**B**) diagram of a monolayer of cell culture grown on BC.

BC structurally resembles natural ECM [120], therefore, BC has been believed to be a promising material for tissue engineering scaffolds [121]. The BC products have been approved by the FDA for use as tissue replacement due to their low endotoxin levels (less than 20 EU per device) [122]. BC scaffolds (Figure 4) have been shown to have the potential to serve as a viable material for chondrogenesis applications due to their ability to successfully regenerate cartilage using human mesenchymal stem cells [123]. In vivo studies consistently demonstrate that BC membranes or scaffolds are typically well tolerated by host tissues after implantation, with no significant adverse effects [124]. Several studies have shown good in vivo biocompatibility of BC-based scaffolds, giving the material potential for use as a scaffold in tissue engineering [21,117,125,126,127].

While the suitability of BC as a raw material for the fabrication of tissue engineering grafts has been demonstrated, the native form of BC is deficient in certain fundamental characteristics that are essential for its utilization in tissue engineering. These include limited biocompatibility, which is a prerequisite for effective tissue regeneration, and inadequate mechanical strength, which is a prerequisite for high-strength applications such as bone and cartilage tissue engineering [128]. Moreover, there is currently no research available that investigates the potential for BC to calcify in vivo over a long-term period [129]. The lack of macropores in native BC also limits its widespread use in tissue engineering [117], as calcification is largely dependent on the porosity of the material and the length of exposure. It should be emphasized that while calcification is undesirable, the degree of calcification will vary depending on the tissue into which the biomaterial is to be incorporated. Appropriate porosity can prevent calcification processes by ensuring angiogenesis and adequate nutrient supply to the cells [129]. In addition, BC has strong mechanical properties, although the presence of many pores limits its stress-bearing capacity [12]. Furthermore, there is a possibility of immunological rejection [106]. To overcome these limitations, the most commonly studied strategies include the production of BC-based nanocomposites with bioactive components, such as polymers and nanomaterials [128].

The majority of research studies have focused on modifying the microporosity of BC to produce materials with the desired properties for replacement or regeneration applications [118,130,131]. Collagen was primarily employed in the development of composite materials with BC for tissue engineering [132,133,134,135,136]. In comparison to the use of collagen composites alone, it has been demonstrated that BC-collagen composites facilitate enhanced cell adhesion and proliferation [132]. In addition, modification BC by proteins such as osteopontin and bone morphogenetic protein 2 (BMP-2) can provide a novel alternative to collagen in the guided bone regeneration field [120,137,138]. In several studies, BC–gelatin composite scaffolds were fabricated for bone tissue engineering applications [117,118,139,140]. BC-keratin composite scaffolds [141] were created for skin tissue engineering. In one study, BC was functionalized by recombinant IKVAV peptide for nerve tissue engineering. BC has also been used for nerve regeneration. BC functionalized with recombinant proteins IKVAV-CBM3 (Ile-Lys-Val-Ala-Val fused with cellulose-binding module) and (19)IKVAV-CBM3 increased mesenchymal stem cell adhesion, cell survival, and neurotrophin expression, which promoted neuronal regeneration [142]. A soy protein isolate (SPI) has been compounded on a double-modified BC to provide a new material for urethral reconstruction [143].

### 3.3. Artificial Blood Vessels

The limited availability of veins in the human body and the potential for severe rejection caused by allografts necessitates the development of artificial veins as a replacement. The most commonly employed clinical artificial vessels are currently constructed of expanded polytetrafluoroethylene (ePTEE, Gore-Tex), polyglycolic acid (PGA), and poly-l-lactic acid (PLLA). However, these materials exhibit several inadequacies that facilitate the formation of thrombi and intimal thickening, which must be addressed in the design of new artificial vein models [144]. In addition, when the procoagulant properties of PET (polyethylene terephthalate, Dacron^®^, DuPont de Nemours, Inc., Wilmington, DE, USA) and ePTFE (Gore-Tex^®^, W. L. Gore & Associates, Newark, NJ, USA) vascular graft materials were compared with BC grafts, BC was shown not to significantly induce plasma coagulation. Compared to PET and ePTFE, BC was found to induce the least and slowest activation of the coagulation cascade and is, therefore, considered a potential vascular graft material [145].

BC and its composites are great options for artificial blood vessels [100,144] by promoting neovascularization. A major problem that can arise in the construction of tubular tissue engineering constructs is the attempt to seed the construct with cells in a tubular state, whereas cells in culture adhere much more readily to a flat scaffold [25]. In hemocompatibility tests, BC typically exhibits a low hemolysis rate, and the mechanical properties of BC-based artificial vessels frequently necessitate enhancement. Modifications of the BC surface through chemical functionalization, in addition to alterations in the manufacturing process, can influence the optimization of BC properties. BC has been functionalized with several macromolecules, including peptides, proteins, and polysaccharides, to improve its hemostatic characteristics. Amino acids have varying electrostatic and hydrophobic characteristics, allowing them to interact with platelets and other blood components via physical and chemical mechanisms to induce hemostasis [146]. To solve this challenge, researchers have mostly focused on making artificial blood vessels with the desired properties and have examined the fermentation procedures and conditions that influence the production of BC tubes [144].

The team of Dieter Klemm was the first research organization to investigate and apply artificial vascular substitute obtained with biomaterials from BC [147] and described a clinical product named BActerial SYnthesized Cellulose (BASYC^®^, Jena, Germany) [100,147,148,149]. The use of BC for the creation of vascular grafts has also been the subject of other studies. Various ECM proteins are used to improve the biofunctional properties of BC membranes and to create a functional endothelial layer [146]. BC/fibrin composites have been developed for the fabrication of artificial blood vessels. However, an investigation of BC/fibrin composites revealed only a slight increase in mechanical properties over those of native BC [150]. In another study, Andrade et al. used BC modified with a recombinant CBM-2 protein and an adhesion peptide tripeptide Arg-Gly-Asp (RGD) to create hemocompatible material [151]. The incorporation of functional peptides usually facilitates protein interaction of ECM by acting as docking sites [152]. In a study conducted by Leitão et al., a novel graft material was created from unmodified, small-caliber, minimally processed BC. The graft’s luminal surface had a similar topography to native vessels. Neovascularization and endothelialization of the graft resulted in the restoration of patency within one month [153].

### 3.4. Cell Culture System

Three-dimensional culture systems are gaining increasing popularity due to their capacity to more effectively mimic tissue-like structures than monolayer cultures [154]. Three-dimensional cell culture models are employed in the prediction of responses to anticancer treatments. In order to accurately and successfully predict treatment options, 3D cell culture must be capable of mimicking the ECM of cancer cells in an artificial environment [155]. Early studies have shown that using commercial hydrophobic proteins enables the hydrophobization of cellulosic cotton fiber [156], and impregnating zein [157] into BC can increase the hydrophobicity of BC surface as well as enhance cell attachment and proliferation when it is used as cell culture scaffold (Figure 4).

Today, natural and synthetic polymers and their composites are employed in the fabrication of 3D scaffolds for tissue engineering and even 3D cancer cell cultures. Cancer cells within a solid tumor maintain close and continual contact with the ECM [158]. Currently, 3D systems used to research tumor behavior, such as Matrigel^VR^ and Geltrex^VR^, are based on natural ECM components. However, chemical variability resulting from the presence of numerous growth factors and proteins, in addition to batch-to-batch variability in these matrices, may interfere with signaling pathway biological reasoning or drug-induced effect outcomes. Consequently, the development of new biomaterials is essential to overcome the limitations of natural ECM [159].

Many researchers have conducted studies to explore the application of BC as a scaffold for 3D in vitro cancer cell models [160,161]. To mimic the characteristics of the tumor microenvironment, BC-based scaffolds were synthesized and evaluated in vitro for their ability to support cancer cell growth [162]. Modifying the pore structure of the BC scaffold can influence the behavior of cancer cells, thereby representing an effective approach for the design and fabrication of in vitro models for the study of cancer biology, potential application in cancer diagnosis, and the development of cancer treatments [121,163,164]. Some molecules, such as hyaluronic acid, chitosan, and gelatin, have been incorporated into the BC network to enhance the mechanical strength, cell adhesion, and cell growth properties of composite scaffolds [155,164,165,166,167].

One of the drawbacks of using BC to make small 3D spheroids is the millimeter scale and low precision of manual fabrication. To produce smaller or more precise shapes from BC-based engineered living materials, it is proposed to use molds or recently developed 3D printing methods for bacterial cultures, such as the «functional living ink» (FLINK) method or a hybrid approach in which a 3D printer is programmed to dispense BC spheroids precisely with different functionalizations [168]. Another challenge of using BC for 3D culture is that it is an uneven three-dimensional substrate for cell attachment with limited integrin binding sites, and cells did not adhere well to BC without surface modification. This can affect cell motility and migration on the surface, as cells migrate toward each other and form huge aggregates that spread in three planes, in contrast to cells cultured on conventional cell plastic, which spread evenly across the surface with no evidence of migration. Morphological variations of individual cells were also observed. To improve cell adhesion and cell proliferation, BC can be modified with the use of different porogens [169] or biodegradable polymers [170] to develop 3D biomimetic scaffolds with interconnected macropores and nanofibrous structure, mimicking the physical structure of ECM. The development of simple, effective physicochemical techniques for surface modification of BC with alternative ECM, growth factors, or other materials could enhance its biocompatibility and biodegradability in vivo [128].

In order to model the tumor microenvironment, it was synthesized and studied BC/gelatin hydrogels as scaffolds for the human breast cancer cell line MDA-MB-231 in vitro culture [171]. BC hydrogel was used for the immobilization of a laminin peptide (IKVAV) to mimic human melanoma cells’ microenvironment and to evaluate the influence of the microstructure and modified chemical surface properties of the resulting matrix [159]. Recently, the BC matrix has been proposed as a tool for trapping and localizing tumor cells within a predetermined region that can be targeted with therapy. The BC scaffold is placed at the tumor site after excision to attract and trap residual cancer cells. Once the cells are immobilized on the BC scaffold, they can be killed by targeted treatment. For example, the chemoattractant human serum albumin (HSA) has been used to capture cancer cells [25]. Biofunctionalized BC scaffold was created for cell replacement therapies in Parkinson’s disease. Human embryonic stem cell-derived progenitor cells were cultured on BC with growth factors and laminin that were covalently functionalized to the surface via silanization [172].

### 3.5. Targeted Drug Delivery System

**Figure 5:**
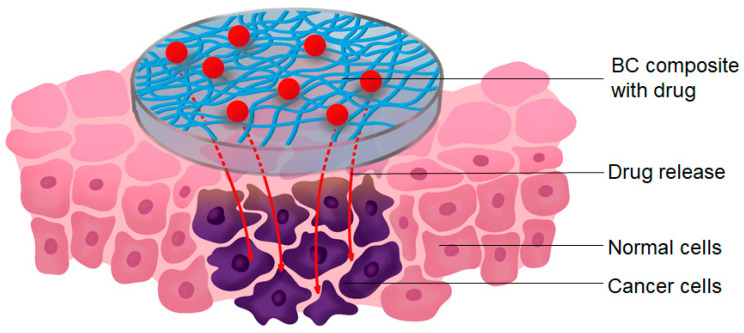
Schematic of the local treatment of cancer cells with a BC loaded with a drug.

The development of a targeted drug delivery system has the potential to markedly enhance the therapeutic efficacy of drugs. Antibodies represent one of the most commonly utilized targeting molecules [173]. BC-based drug delivery systems have been a subject of considerable interest recently [32,174]. BC is often mixed with other materials to provide controlled drug release mechanisms. It has been shown that the properties of BC composites can be changed [175], allowing these composites to be tuned for use in a wide range of biomedical applications requiring varying drug release rates. BC has been shown to be a viable substance for long-term drug release, making it an excellent carrier for cancer therapy [174]. A key advantage of using a drug carrier such as BC is that it allows for controlled and localized treatment, which can increase drug concentrations at the tumor site [25] (Figure 5).

Two transdermal delivery systems for immune checkpoints (anti-CTLA-4 antibody [32], 131I-αPD-L1 antibody [176]) to treat melanoma cells have been developed based on BC. These novel approaches offer distinct advantages that can be leveraged to enhance the efficacy of immunotherapy. The controlled release of antibodies via delivery systems such as BC represents a highly attractive strategy for reducing the systemic dissemination of antibodies and potentially mitigating the adverse effects associated with checkpoint therapy [32].

A recent study has indicated that the BC may be an appropriate nanocarrier for developing vaccines for aquatic animals. The use of carboxylated BC by 2,2,6,6-tetramethyl-1-piperidinoxyl (TEMPO) oxidization was employed to conjugate ribavirin to the NbE4 nanobody, with the objective of developing a drug system against the largemouth bass virus [173]. In another study, a system for delivering cyano-phycocyanin to the gastrointestinal tract was developed based on BC nanocrystals (BCNC). This system has been shown to protect phycocyanin release from degradation by gastric fluid until phycocyanin reaches target sites [177].

Although researchers have reported encouraging results with the use of BC-based materials for drug delivery, most studies are still in their early stages. Some research focuses solely on in vitro drug release [98], while others primarily use animal models. As a result, more clinical trials are needed to ensure the safety and efficacy of BC-based materials before they can be commercialized. In addition, most of the drugs used in BC-based drug delivery systems are model drugs. More sophisticated drugs used to treat specific diseases are unlikely to be useful due to uncertain interactions between BC and other treatments. To improve the efficiency of drug delivery, ensure biocompatibility, and adjust the hydrophilicity/hydrophobicity of BC, it is necessary to optimize the composite formula based on BC; some drugs need additional research and integration of 3D printing [174].

### 3.6. Enzyme Immobilization

Enzyme immobilization reduces operating costs, extends enzyme life, increases enzyme stability, and facilitates recovery and reuse [178]. The immobilization of the enzyme on BC ensures high contact between the substrate and the immobilized enzyme due to the fast diffusion of substrates and products into the aqueous solution through the network of BC nanofibrils, which ensures high turnover of the enzyme [179].

The most common and most effective way to immobilize enzymes is to attach them to highly activated supports. The enzyme’s primary amino groups are good at reacting with activated supports, and they do not need to be activated [180]. The primary amino groups in enzymes can be classified as either highly reactive at neutral pH or low reactive at neutral pH due to the high pK (10.5) of lysine residues. Although the less reactive lysine residues in this area are 1000 times less reactive than the single N-terminus, immobilization of the enzyme through this region is necessary to achieve the best multipoint immobilization [180].

One of the disadvantages of utilizing both BC and the majority of carriers is the necessity to activate them prior to immobilization, as well as the relatively low efficiency of immobilization, especially with BC [14]. Furthermore, immobilization may result in enzyme inactivation or a low initial activity of the immobilized enzyme. This may be due to limited diffusion of the substrate and products through the cellulose matrix in which the hybrid protein is embedded. The enzyme activity may also be affected by the method of BC drying. This may affect the access of water to the enzymes bound in the inner parts of the membrane, thereby changing their hydrolytic activity [178].

One way to alter the BC matrix used for enzyme immobilization is to add substances such as carboxymethylcellulose (CMC), chitosan, alginate, and lignin derivatives to the culture medium. Similarly, modifying the drying conditions of BC membranes can change the physical and chemical characteristics of BC, as it impacts the membrane’s porosity and its capacity to adsorb enzymes [13,14,15,16]. These treatments significantly enhance the potential for using BC as a carrier of enzymes or other active compounds [181].

To date, both unmodified and modified BC have been successfully employed for the immobilization of numerous enzymes, including papain [110], lysozyme [111], lipase [14,182,183,184], β-galactosidase [178], horseradish peroxidase (HRP) [185], superoxide dismutase (SOD) [4], glutamate decarboxylase (GAD) [45], laccase [108,186], lecitase [181], urease [187], L-asparaginase [188], and others. The enzymes were immobilized using unmodified BC and modified by chitosan BC hydrogel beads, as well as preactivated BC with glutaraldehyde or oxidized BC with sodium periodate.

## 4. Methods of Protein Immobilization on Bacterial Cellulose

**Figure 6:**
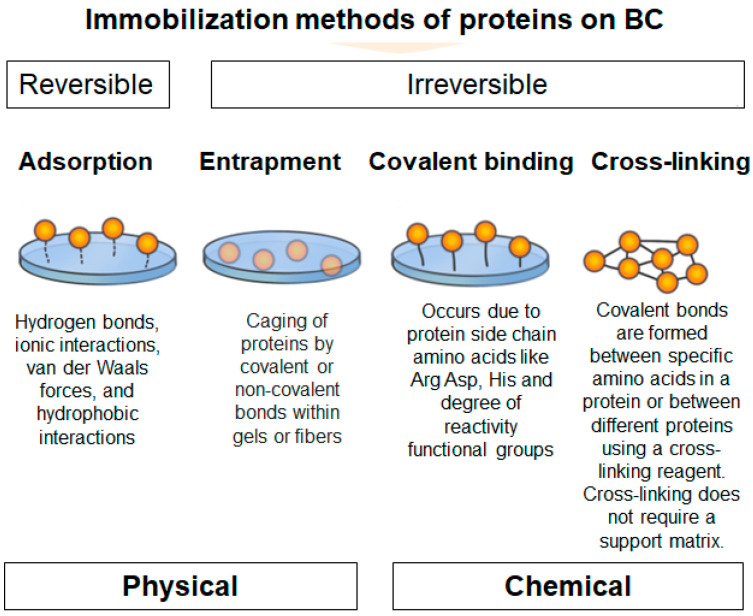
Schematic illustrating protein immobilization methods on BC. Physical methods include adsorption and entrapment. Chemical methods include covalent binding and crosslinking.

The latest advances in nanostructured carrier material and immobilization technique development now allow for precise protein immobilization, including on BC [189]. Protein immobilization on BC can be achieved through a number of different methods, including covalent binding, adsorption, crosslinking, and entrapment (Figure 6).

Physical immobilization techniques utilize physical interactions to stabilize proteins and enzymes onto cellulose substrates. This approach frequently results in only minor alterations to the protein [10,178]. Compared to other immobilization techniques, physical immobilization methods are relatively straightforward and cost-effective. The primary physical immobilization methods are entrapment and adsorption [10].

Among protein and enzyme immobilization methods, adsorption is the simplest. Adsorption is usually accomplished by hydrophobic interactions and salt bridges, and protein function is largely preserved because the bond between the support and the protein or enzyme is minimal [190]. The degree of protein adsorption on BC depends on the characteristics of the native form of BC, such as the porosity, the degree of cross-linking of the nanofibrils, and other material properties, depending on the culture time and the composition of the medium [191]. The efficiency of protein immobilization on BC can reach over 93,5% with physical adsorption [14]. Recently, a protein imprinted material based on a BC composite carrier and a metal–organic framework, zeolite imidazolate framework-67 (ZIF-67), was developed for the isolation of bovine serum albumin. This material exhibited ultra-high adsorption capacity (1017.0 mg/g), excellent recognition (IF = 5.98), and fast adsorption equilibrium time (50 min) [16].

The cellulose matrix is one of the most optimal substrates for the immobilization of enzymes by entrapment [178]. Entrapment methods do not place proteins directly on the surface of their supports, in contrast to adsorption approaches. Instead, they are physically entrapped within the polymer matrix. Entrapment protects proteins and enzymes from hostile conditions and improves their stability [192]. Nevertheless, due to the weak interactions involved, physical methods often result in enzyme degradation during washing, which can lead to loss of functionality. It is, therefore, essential to employ enzyme purification as a preliminary step in such cases [178].

Protein immobilization by crosslinking is the process of chemically joining two or more molecules by a covalent bond [193]. The protein is typically adsorbed to the nanocarrier and then cross-linked using a bifunctional agent like glutaraldehyde [194]. The combination of two groups in a single protein results in intramolecular cross-links, which reinforce the protein’s tertiary or quaternary structure. Intermolecular cross-links are formed when groups of two distinct proteins bind together, creating a stable protein-protein connection [193]. This method improves enzyme stability and reduces leakage, which is common with non-covalently attached enzymes on supports [194].

Covalent bonds between protein functional groups and support materials are often used for immobilization [192]. In this approach, proteins are securely attached to the surface by covalent bonds [195]. In addition, covalent bonding has a high loading efficiency [196], which is not possible with other methods [8]. The covalent attachment process involves the binding of amino acid residues (i.e., –NH2, –COOH, –SH) to support matrices [48]. Hydroxyl groups on a cellulose surface may interact with proteins, but this is not sufficient for the covalent immobilization process. Therefore, additional functionalization procedures must be conducted to achieve a robust covalent immobilization. The incorporation of functional groups into cellulose surfaces that may react with amino acid residues was accomplished through modification of the cellulose matrix [10].

There are two methods for functionalizing cellulose. The first method entails the addition of amino groups to the surface of cellulose, thereby enabling the formation of a reactive complex with the carboxylic acid groups of amino acids. The second approach involves the introduction of an aldehyde, carboxyl, or epoxide moiety that can engage in a reaction with the amino (–NH₂) group. Another strategy for immobilizing proteins and enzymes on cellulose is to use protein carboxyl groups to react with matrix functionality, including the amino groups present in the cellulose matrix. Activated groups like carboxyl and aldehyde can attach to protein amino groups. To produce aldehyde or carboxyl groups, several chemical methods were employed to oxidize the hydroxyl groups of cellulose [10].

Covalent binding is the most stable approach for enzyme immobilization on cellulosic supports, which can increase the activity and thermal stability of the immobilized enzyme [63,197]. However, protein immobilization by covalent coupling to polymeric materials offers several outstanding advantages for a wide variety of applications, yet coupling techniques are typically limited by their high cost or complexity [198]. The efficiency of protein immobilization by covalent binding to BC may vary depending on the method used. The immobilization efficiency of conjugated recombinant human osteopontin, coupled with the poly(acrylic acid) (PAA)-grafted BC, has been determined to be 97% [15]. However, the strong binding has implications as the enzymes are chemically modified and lose some of their catalytic activity [8]. The use of chemical coupling agents can potentially inactivate the protein or enzyme [10,178]. In some cases, chemical modification of BC (for instance, by diphenyltetrazole) may lead to ineffective protein conjugation [178,199]. Examples of BC modification for protein immobilization in biomedicine will be discussed in detail in the next chapter.

## 5. Ex Situ and In Situ Modifications of Bacterial Cellulose for Protein Immobilization

Despite the development of new BC-based composites, there are still obstacles to overcome before BC can be fully utilized as a biomedical material. The main issues that need to be addressed are optimizing culture conditions to control the porosity of the BC scaffold, incorporating functional groups into the BC matrix, and increasing the degradation rate of BC to suit specific applications [99,200]. Furthermore, the utilization of native BC presents a challenge in the form of dehydration during the drying process. To prevent dehydration, BC can be modified by various methods to achieve the desired properties [201]. Although BC has a high degree of crystallinity and a single functional hydroxyl group, providing low solubility and limiting its application, BC contains a high concentration of hydroxyl groups on its surface that can be modified. The focus is on modified BC with multiple functional groups that exhibit diverse surface properties, such as lipophilic-hydrophilic properties and magnetic and optical capabilities, along with a regulated specified functionalization pattern [62].

**Figure 7:**
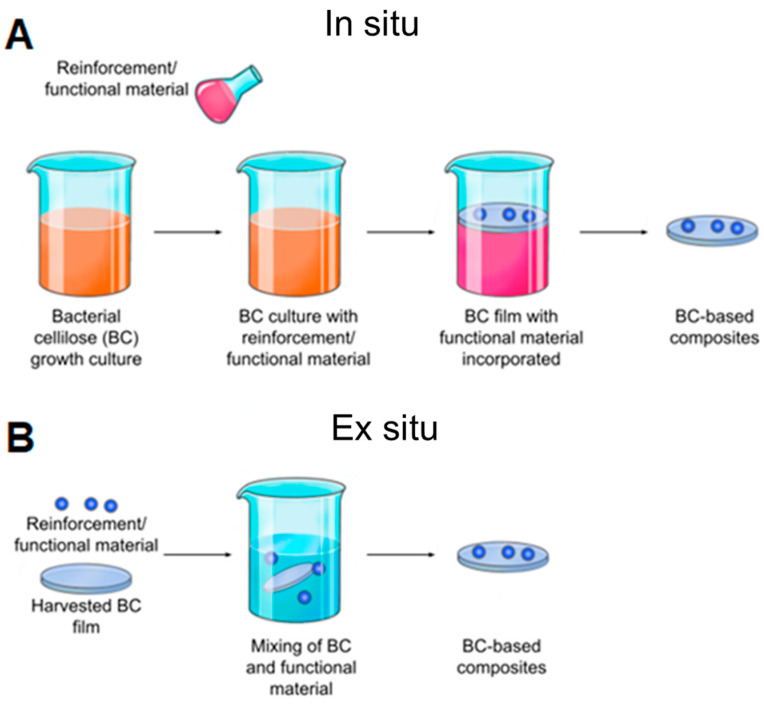
Schematic representation of BC modification methods. (**A**) In situ modification involves changing the composition of the culture medium, typically through the addition of other materials. (**B**) Ex situ modification is a process whereby BC is treated with chemicals or absorbed by other materials after the BC membrane has been formed in culture.

To achieve these goals, BC has been modified in a variety of ways, including chemical modifications (changes in chemical structure and functionality) and physical modifications (changes in porosity, crystallinity, and fiber density). In general, there are two main approaches to implementing these changes: ex situ and in situ [202] (Figure 7).

An ex situ modification is the most common modification of BC [203]. Ex situ modification of BC occurs when an exogenous macromolecule interacts primarily with the BC surface and can penetrate through membrane pores. Changes in the physical-chemical parameters of the BC composite are determined by the degree of exogenous molecule incorporation into the membrane [98]. The key benefits of ex situ modification of BC include a wide range of composite synthesis techniques, the ability to utilize liquefied and suspended materials [204], the maintenance of BC’s primary structural characteristics [11], and the avoidance of issues associated with the incorporation of antimicrobial materials [205].

However, the size and nature of the exogenous molecule (reinforcing material) pose the greatest challenge to ex situ composite synthesis. Only submicron to nanoscale materials can be implanted into the BC matrix. This is because larger particles cannot pass through the BC pores, and hydrophobic materials cannot combine with BC. In addition, the structural arrangement of the BC fibrils is not always regular, so penetrating materials may not be uniformly distributed throughout the BC matrix [12]. In addition, unless a covalent chemical modification is performed, the interaction between the BC membrane and the exogenous molecule is weaker compared to the in situ process [98]. The influence of many modifications on environmental and host physiological conditions is still not fully known and, therefore, requires further investigation [174]. For example, treatment of cells with NaIO_4_ has been shown to result in the formation of free surface aldehydes that lead to cross-linking between cells via a Schiff base and cause cytotoxicity [206].

There are two forms of ex situ modification: chemical and physical. Physical ex situ modification is usually achieved by physical absorption—a porous BC matrix can be filled with solutions or particle suspensions—the presence of hydroxyl groups on the cellulose chains often results in strong hydrogen bonding between the BC molecules and the absorbed molecules to achieve modification [80,202,207].

In the case of chemical ex situ modification, a reaction with chemicals to change BC’s chemical composition takes place. Since the chemical nature of BC is cellulose, it can be phosphorylated and then modified by graft copolymerization or crosslinking [208]. Carboxymethylation [209], acetylation [203], phosphorylation [210], esterification [211], and other graft copolymerization and crosslinking processes on the BC surface have produced a wide range of BC derivatives with unique structures and properties.

Chemical modification of the BC structure disrupts the ordered crystal-forming hydrogen bonds and increases the water solubility of even hydrophobic derivatives [25]. Incorporation of additional functional groups into the BC structure can impart to BC hydrophobicity, ion adsorption capacity, and optical properties while retaining the characteristic three-dimensional nanostructure and superior mechanical properties of BC. For example, oxidation under moderate aqueous conditions can preserve the crystallinity and size of BC. Recently, acetylation of BC via a non-swelling reaction mechanism has been reported to increase its dispersibility and compatibility in various solvents or matrices suitable for nanocomposite fabrication [212]. The hydrophobicity of the acetylated surface is beneficial for maintaining a large surface area after drying from water and also makes the microfibrils compatible with other hydrophobic materials [62]. The surface modification of BC matrices can improve drug loading and release capabilities. The results indicated that surface modification of BC matrices can alter the surface properties [207]. Most BC modifications are aimed at improving its applicability and performance in a variety of applications (Table S1 in Supplementary Materials).

Biosynthetic (in situ) modification of BC represents an environmentally friendly method that is also simple and cost-effective. This process can be combined with various different chemical additives present in the culture solution to create scalable nanocomposites [213]. In contrast to ex situ modification, in situ modification is relatively straightforward to perform and exhibits uniformity in modification effect. The application of in situ modification of BC presents certain challenges, including the precipitation of additional compounds, the inability to successfully incorporate reinforcement materials into the pellicle [214], and the stringent conditions required for bacterial growth [203]. Although in situ BC modification is commonly used in tissue engineering applications, the stringent microbial fermentation conditions limit the entry of a wider range of additives. Other concerns with the in situ modification process, such as interactions between externally added additives and BC fibril formation, as well as structure controls of BC nanofibers, need to be addressed [202]. In addition, there are limitations in the synthesis of BC composites with antimicrobials [215], the use of BC composites produced by the agitation technique [216], and the disturbance of the main structural features of BC [217]. By incorporating liquid and nanoparticles into the structural matrix of the prepared BC, some problems associated with the in situ synthesis of BC composites can be solved [12].

### 5.1. Ex Situ Bacterial Cellulose Modification

#### 5.1.1. Native Bacterial Cellulose

**Figure 8:**
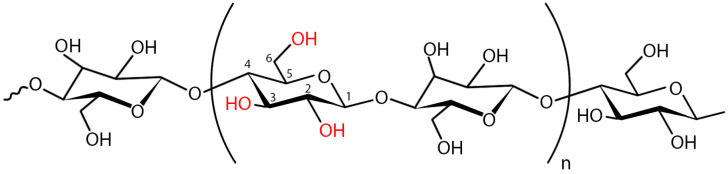
The structure of repeating dimer units of the native (unmodified) BC. The dimer demonstrates the intra-chain interactions in cellulose. The numbers indicate the positions of carbon atoms in the glucose molecule. Hydroxyl groups are shown in red. The two most accessible hydroxyl groups on cellulose for substitution are OH-C6 and OH-C2, while OH-C3 is less accessible. *n* = degree of polymerization.

Native BC (Figure 8) exhibits superior mechanical strength and stability [150] and high water absorption capacity [218] in the wet state. Implants created from native BC exhibit gradual, non-enzymatic hydrolysis, which is determined by the chemical composition of the main chain and side groups, aggregation state and shape, hydrophilic-hydrophobic balance, surface, and other variables. This process is of utility in certain applications [219], for example drug delivery or tissue engineering. It was shown that unmodified BC did not affect the antibody binding efficacy [32]. Furthermore, unmodified BC membranes do not possess any inflammatory or immunogenic properties [20,32,65,220]. A grafted, native BC membrane serves as a physical barrier, reducing pain and the risk of infection, and allows drug delivery to the wound [221]. In contrast, unmodified BCs are characterized by the immediate release of drugs, regardless of the solubility of drugs in water and the dose [207].

There are several studies on the use of native BC as a carrier for proteins. A macroporous BC hydrogel was developed for wound healing through a process of physical punching with a stainless mold to generate uniform holes with a size of 0.5 mm in diameter, separated by a constant distance of 2 mm. The generation of the macroporous BC hydrogel was achieved by direct layering of the BC hydrogel on top of an alginate solution, with CaCl_2_ promoting the integration of the alginate into the BC. Then, BC hydrogel was immersed in ECMs (collagen, elastin, and hyaluronan) and growth factors (B-FGF, H-EGF, and KGF). The modified BC hydrogels were shown to be biodegradable under physiological conditions, and growth factors were gradually released. The H-EGF and collagen-modified BCHG were found to support the growth of human skin fibroblasts [70].

A BC–sericin composite was developed for wound healing. For this purpose, BC was impregnated with a sericin solution for 24 h with stirring. The resulting composites exhibited a homogeneous, highly porous structure, a smaller pore size, and a high swelling capacity when compared to BC. However, no significant difference was observed between the effect of BC and the BC–sericin composite on the behavior of keratinocyte cells during cultivation. Additionally, no significant changes were noted in the thermal and mechanical stability of the BC network after the addition of sericin [105].

Modification of native BC with soybean isolate protein was utilized for wound healing treatment. The surface roughness and hydrophilicity of BC–soy protein composites are reduced compared to native BC, and soy protein could be stably released. The resulting composites promoted improved adhesion and proliferation of normal human dermal fibroblast culture and type I collagen expression in vitro compared to the control. At the same time, cell viability increased by almost 50% compared to BC. The composites promoted accelerated wound healing (17 days versus 21 days for wound treatment by control). In addition, BC–soy protein composites stimulated collagen deposition (five times higher than the control), enhanced angiogenesis and hair follicle regeneration, and helped reduce scarring and skin inflammation in rats [13].

Nisin-loaded BC membranes were developed to preserve food quality and inhibit the growth of microbial contaminants. When the antimicrobial activity of the resulting membranes was evaluated by minimum inhibitory concentration and agar diffusion assay using *Staphylococcus aureus* (*S. aureus*), *Escherichia coli* (*E. coli*), and *Pseudomonas aeruginosa* (*P. aeruginosa*), it was shown that nisin in combination with EDTA exhibited significant antimicrobial and antioxidant activity against *S. aureus* (MIC was 15.63 μg/mL) and *E. coli* (MIC was 31.25 μg/mL). No antimicrobial activity was observed against *P. aeruginosa* [113].

Two distinct methodologies were employed to functionalize BC with the antimicrobial peptide ε-poly-l-lysine. The first strategy involved adding ε-PLL to CMC-functionalized BC membranes using EDC (N-(3-dimethylaminopropyl)-N’-ethylcarbodiimide hydrochloride) and NHS (N-hydroxysuccinimide) to form the amide bond [190]. The second strategy involved directly crosslinking ε-PLL with the BC structure using carbodiimide chemistry to form a stable interpenetrating network. Both techniques yielded membranes that were biocompatible with human fibroblasts and capable of inhibiting *S. epidermidis* development upon contact [222].

Bacteriocins from *Lactobacillus sakei* subsp. sakei 2a (*Lb. sakei* 2a) strains were immobilized on BC membranes to enhance their antimicrobial activity against *Listeria monocytogenes* (*L. monocytogenes*, foodborne pathogen). Immobilized bacteriocins were significantly (*p* < 0.05) more effective in controlling pathogen growth than the free bacteriocins throughout the study period [115]. In another study, commercial laccase and silver nanoparticles were physically adsorbed onto BC for wound dressings. The specific activities of immobilized and free laccase were similar. However, the value of the Michaelis–Menten constant (Km) for immobilized laccase (0.77 mM) was almost twice that of the free enzyme. The antimicrobial effect of laccase on medically relevant strains was 92% (*S. aureus*) and 26% (*E. coli*), while the composite had no cytotoxicity on fibroblasts [108].

To enhance the biocompatibility and osteoinductivity of BC, Huang et al. developed a porous BC scaffold modified with gelatin and coated with hydroxyapatite. Gelatin was introduced into the surfaces of BC nanofibres by physical adsorption or via the procyanidins crosslinking technique. The results demonstrated that procyanidine crosslinking led to a greater improvement in Young’s modulus and maximum load of BC scaffolds compared to physical crosslinking. A notable increase in mechanical properties was observed in the order of BC, BC/gelatin, BC/procyanidine/gelatin, and BC/procyanidine/gelatin/hydroxyapatite scaffolds. The BC/procyanidine/gelatin/hydroxyapatite scaffold exhibited superior adhesion, viability, proliferation, and osteogenic differentiation of human bone marrow stromal cells. In vivo studies in nude mice and rabbits demonstrated that the BC/procyanidine/gelatin/hydroxyapatite composite exhibited the most favorable osteogenic properties [117].

A recent study demonstrated that BC could serve as a carrier of BMP-2 (an osteoinductive cytokine) for bone regeneration. The rabbits treated with the BC/BMP-2 composite exhibited significantly more newly formed bone than the other groups. The new bone was found to have a markedly higher number of PCNA-positive cells compared to sites away from the composite. After eight weeks, the composite exhibited continuous release of BMP-2. Additionally, no rabbits showed any noticeable inflammation, and no capsules developed around the BC or the BC/BMP-2 combination [138].

A novel keratin-containing BC nanocomposite with the potential to enhance skin fibroblast adherence to the BC surface was developed and characterized by Lin et al. The BC-containing keratin composites were obtained through both in situ and ex situ modification. In comparison to native BC and in situ modified BC/keratin, the viability of keratinocytes and fibroblasts on post-modified BC/keratin nanocomposites was found to be higher. In vitro cell culture studies have demonstrated that cutaneous fibroblasts have good attachment and proliferation on post-modified BC/keratin nanocomposites [141].

Different recombinant bioactive peptides IKVAV, (19)IKVAV, and RGD were fused to a CBM3 to functionalize BC surfaces to promote neuronal and mesenchymal stem cell (MSC) adhesion. It was demonstrated that there was an improvement of almost 100% in cell adhesion for PC12 cells and 30% for MSCs. The RGD-CBM3 protein also exhibited the capacity to enhance the adhesion of N1E-115 and mesenchymal cells. Additionally, the IKVAV-CBM3 facilitated the release of neurotrophic factor (NGF) secreted by MSCs into the culture medium [142].

One study utilized native BC, which was dissolved in N-methylmorpholine N-oxide (NMMO), followed by the addition of porogen (sodium chloride) and then gelatin to create a composite that can be applied in tissue engineering. The resulting composite had high porosity and rapid swelling. In vitro biological tests demonstrated that animal fibroblast cells (NIH 3T3) adhered well and proliferated well on the BC–gelatin composite scaffolds. Increased expression of metalloproteases indicated that long-term incubation of cells can lead to the formation of ECM within the resulting 3D scaffolds [118].

Surface modification of native BC was performed with tripeptide Arg-Gly-Asp (RGD) fused to a cellulose-binding module for the development of hemocompatible material. The RGD is found in many adhesive plasma and ECM proteins and has been shown to improve cell adhesion. The plasma recalcification time and whole blood clotting results demonstrated that BC did not interfere with the coagulation process. A significant amount of plasma protein was adsorbed to BC fibers, and the adsorption of plasma proteins to the BC fiber surface did not affect its protein structure. Human microvascular endothelial cells grown on RGD-modified BC developed a confluent cell layer, which inhibited platelet attachment [151].

Human serum albumin (HSA) was chosen as a model protein to study the loading of chemoattractants onto and released from BC membranes in the F98 rat glioma model. The BC membrane was found to confine F98 tumor cells, preventing their migration once attached to the membrane surface (even in the presence of an attractive medium in the environment). F98 cells trapped on BC remained viable and retained the ability to grow, adopting a spheroid pattern of growth [223,224].

BC was studied as a carrier for antibody delivery. To investigate the release of antibodies in vitro and in vivo, BC was loaded with a model IgG antibody and an anti-CTLA-4 antibody. In vitro experiments demonstrated that IgG was released within 24–48 h. Experiments on cell cultures indicated that BC did not have a cytotoxic effect on the M39 cell line and did not cause activation of dendritic cells. In vivo investigations in serum demonstrated that BC hydrogels significantly reduced the levels of IgG and anti-CTLA-4 antibodies when compared to the levels of antibodies in PBS. The antibodies loaded in the BC retained their binding capacity, as compared to antibodies from a stock solution, after 14 days of implantation [32]. Additionally, BC films were utilized to facilitate the delivery of L-asparaginase to melanoma cells. L-asparaginase was immobilized via physical adsorption. The maximum adsorption of L-asparaginase was observed among BC films grown for 96 h, reaching 84.5 ± 5.7%. Uveal melanoma cells (A875) demonstrated sensitivity to L-asparaginase, with an IC_50_ value of 0.03. L-asparaginase immobilized on BC caused the death of over 90% of tumor cells after 72 h [188].

BC has also been used to immobilize superoxide dismutase (SOD) to increase its stability at high temperatures and protect fibroblasts against oxidative damage. The results demonstrated that the immobilized SOD was stable at pH levels ranging from 4 to 8, with approximately 70% of the remaining activity. In contrast, the free SOD lost 70% of its initial activity. At temperatures ranging from 25 to 40 °C, the immobilized SOD retained more than 80% of its residual activity. The residual activity of immobilized SOD exhibited a gradual decline from 40 to 45 °C, reaching 30% of the initial activity at 50 °C. In comparison, the activity of free SOD demonstrated a precipitous decline at temperatures above 40 °C. The fibroblast cells that were incubated with BC/SOD and subsequently treated with hydrogen peroxide demonstrated a cell viability of 78.46%, which was higher than that observed in the induced fibroblast cells [4].

Unmodified or dry BC membranes were used to immobilize wild-type β-galactosidase and β-galactosidase with a thermostable module CBM2 (TmLac). The CBM2 domain allows direct immobilization of cellulose substrates with high specificity. The binding efficiency of the TmLac-CBM2 hybrid was similar to hydrated BC and freeze-dried BC. The TmLac-CBM2 protein bound to BC more strongly at pH 6.5 than at pH 8.5 and with high specificity compared to the wild-type enzyme. The CBM2 module fused to the enzyme provided a stable attachment to cellulose at 75 °C. The efficiency of lactose hydrolysis was similar between the three forms of β-galactosidase. Enzyme recycling was limited by the instability of the β-galactosidase module, whereas the attachment of CBM2 to cellulose was stable even at 75 °C for 3 h [178].

#### 5.1.2. Bacterial Cellulose Nanoparticles

BC has a wide range of applications in the biomedical field. However, the usage of BC in the form of films or membranes, which is produced by static culture fermentation, limits its applicability [225]. Furthermore, BC scaffolds have some other drawbacks, such as a lack of antimicrobial properties (for use as dressings) and modest mechanical strength [62,226].

**Figure 9:**
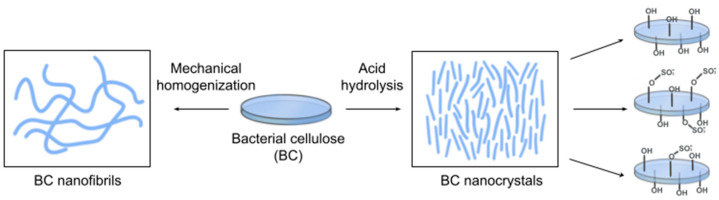
Schematic of methods for obtaining BC nanofibrils (BCNF) and BC nanocrystals (BCNC) from BC for protein immobilization: BCNF production from BC by homogenization (left); BCNCs production from BC by acid hydrolysis with different types of acid surface modification (right). The most commonly used acids are hydrochloric acid (HCl), sulfuric acid (H_2_SO_4_), and a mixture of HCl and H_2_SO_4_.

Nanoparticles derived from BC can be classified into two categories: BCNCs and BCNFs (Figure 9) [227]. BCNC and BCNF can be obtained from BC using acid hydrolysis and mechanical homogenization, respectively [228,229,230]. Unlike the hydrogel structure of BC in its natural form, BCNC, and BCNF can be dispersed in an aqueous solution and easily incorporated into polymer networks that act as reinforcing agents [231]. These nanoparticles have different sizes, shapes, and properties [227].

##### Bacterial Cellulose Nanofibrils

BCNFs make up over half of the research on nanocellulose and have been a European bioeconomic priority since 2008 [232]. BCNFs, like CNFs, are flexible, nanosized fibrils with a high aspect ratio. They can form strong, entangled, and disordered networks. BCNFs are long and flexible nanofibers that contain both crystalline and amorphous areas. They consist of fibrillar elements that are 10–50 nm wide and several micrometers long [227]. The BCNF solution is stable, which enhances the versatility and performance of this cellulose material [225]. BCNFs interact with other inorganic particles or biomass components (such as polyphenols, polysaccharides, or proteins) to form unique complex structures [233]. BCNFs are considered safe biomaterials in accordance with the FDA’s Generally Recognized as Safe (GRAS) standard [122].

In a recent study, BCNF was utilized to immobilize lysozyme through a process of physical absorption. After immobilization, lysozyme activity decreased by approximately 12%, but storage stability was improved, and immobilized lysozyme retained more than 70% of its original activity after nine cycles of use. Immobilized lysozyme showed enhanced antimicrobial activity against *S. aureus*, *E. coli*, *L. monocytogenes*, *Yersinia entrocolitica*, *Aspergillus niger*, and *Saccharomyces sereviseae* [111].

A BCNF–zein composite with controlled surface hydrophobicity was created for tissue engineering by Wang et al. The use of zein was based on the ability of zein to self-assemble into various microstructures upon solvent evaporation, as well as its good biodegradability and high biocompatibility. First, BCNF was immersed in zein solutions with gentle stirring, then self-assembly of zein molecules occurred under evaporation, followed by hot pressing. An increase in surface roughness and hydrophobicity of BCNF was observed with the addition of zein at low concentrations (5 mg/mL), while the opposite effect was observed with a higher zein concentration (2%). The incorporation of zein on the surface of BCNF did not significantly alter the internal structure and mechanical properties of BCNF. Compared to pure BC, BCNF–zein composites showed significantly increased adhesion and proliferation of fibroblast cells [157].

BCNFs have been used to construct a delivery system for radiotherapy and immunotherapy in the treatment of metastatic cancer. To address the challenges associated with the clinical application of immune checkpoint blockade and the nonspecific distribution of radioisotopes, an injectable suspension of 131I-labeled antibody against programmed cell death ligand 1 (αPD-L1) immobilized on BC was developed. The resulting composites were targeted specifically to the tumor and stimulated the immune response to achieve specific cancer radioimmunotherapy. The biocompatibility, long-term antibody retention, and immunostimulatory effects of 131I-αPD-L1/BC were confirmed in vitro and in vivo. After long-term treatment with 131I-αPD-L1/BC, T cells in lymph nodes were polarized to CD8+ CTL, which killed cancer cells in the tumor. Radioimmunotherapy prevented cancer from spreading in a breast cancer model [176].

BCNF-chitosan composite hydrogel beads were prepared as scaffolds for the immobilization of *Candida rugosa* lipase. To prepare BC-chitosan hydrogel beads, chitosan was dissolved in 1-ethyl-3-methylimidazolium acetate. BC powder was added, and the mixture was stirred and dried. The amino groups of chitosan were converted to aldehyde groups after treatment with GA. Lipase was immobilized by crosslinking GA or physical adsorption. Cross-linked lipases showed higher stability than adsorbed and free lipases. After 30 min incubation at 60 °C, the residual activity of BC2 was 76%, while free lipase retained 43% of initial activity. After 10 h incubation, the residual activity of BC2 was 44%, while that of free lipase was 15%. The half-life time of lipase adsorbed on cellulose-chitosan beads was found to be 2.7–3.7 times higher than that of free lipase. The half-life of lipase cross-linked to BC-chitosan beads at 60 °C was 22.7 times that of free lipase [183].

##### Bacterial Cellulose Nanocrystals

BCNCs are rod-like nanoparticles created from BC after selecting and eliminating the amorphous region. They have a high crystallinity and a rigid structure with a length of 100–1000 nm and a width of 10–50 nm [227]. BC can be hydrolyzed using strong acids such as H_2_SO_4_ and HCl to generate a stable solution of BCNC, which provides the material with new functionality [234]. However, acid hydrolysis removes the amorphous portion of cellulose, reducing yield [231]. BCNCs can be used as building blocks for a wide range of applications [234].

Sakacin-A/BCNC conjugates have been developed for use in antimicrobial food packaging. Sakacin-A is an anti-Listeria bacteriocin produced by *Lb. sakei*. The resulting conjugates were found to be stable when incubated in neutral and mildly acidic solutions (pH 5), but Sakacin-A completely dissociated from BCNCs in alkaline conditions (pH 11). The Sakacin-A/BCNCs conjugate-coated samples exhibited superior surface roughness and tensile strength compared to the paper substrate. The antimicrobial packaging was effective in both in vitro and cheese experiments. The paper samples coated with Sakacin-A and Sakacin-A/BCNCs conjugates had similar antimicrobial activity [114].

A 3D-printed scaffold comprising BCNC, gelatin (GEL), polycaprolactone (PCL), and hydroxyapatite (HA) was developed for use in bone tissue engineering. The 3D printing procedure was used to create four different scaffold compositions with 50%, 60%, 70%, and 80% infill rates. The 3D scaffolds with an 80% infill rate exhibited a pore size (~300 µm) that was suitable for bone tissue engineering. These scaffolds demonstrated a uniformity ratio exceeding 90%. The incorporation of BC and HA into the PCL/GEL scaffold enhanced the growth and attachment of human osteoblast cells. The 3D-printed scaffolds exhibited osteoblast cells with large cytoplasmic dendritic structures, which resembled the appearance of osteocytes [140].

In work [182], lipase was immobilized on BC and BCNC. BC and BCNC were functionalized with succinic acid as a linker [235], and the lipase was then conjugated to succinylated cellulose using EDC/NHS. After immobilization, the enzyme retained its activity in both BCNC and BC membrane, and the amount of protein immobilized on BCNC was 2.75 times higher than that in BC membrane. The BCNC was also employed for the immobilization of urease. The immobilized urease demonstrated superior tolerance to changes in pH (5.5–9) and temperature (30–80 °C) when compared to the free urease. Furthermore, the immobilized urease retained approximately 81% and 68% of its initial activity following 15 and 20 cycles of reuse, respectively. It also exhibited significantly enhanced storage stability for 20 days [187].

#### 5.1.3. Crosslinking

Crosslinking is defined as the induction of chemical or physical links among polymer chains [236]. The crosslinking of materials can be achieved through a variety of methods, including physical processes, chemical processes, and enzymatic processes [237]. The chemical crosslinking method makes it possible to obtain an irreversible or permanent hydrogel. The physical crosslinking method can produce a hydrogel that can be reversed since the forces involved are hydrophilic interaction, electrostatic, and hydrogen bonding. Crosslinking improves the thermal and mechanical stability of the matrix and can be tailored to modify the release rate of the incorporated active agents [238].

The abundant hydroxyl functional groups in the BC molecular chains make BC an excellent candidate for modifications by crosslinking [202]. Crosslinking plays a pivotal role in the drying process [68,239], prevents the collapse of 3D network BC in the drying process [163,240,241], and improves water absorption [242]. In this sense, cross-linked samples may absorb water faster than pure samples, and their spongy structure results in a significant variation in surface shape compared to native samples [243]. The chemical structural similarities between BC and plant cellulose make it possible to utilize plant cellulose crosslinkers [163,240,241].

##### Chemical Crosslinking

Chemical crosslinking is the process of incorporating monomers into polymers or joining two polymer chains using crosslinking agents [9,244]. Several different types of crosslinking agents have been used in published studies of cellulose-based hydrogel materials. Typically, when considering crosslinking agents, one is interested in compounds that are capable of forming pairs of covalent bonds, thereby linking polymeric segments together [245].

- Glutaraldehyde

GA is widely used as a crosslinking agent in biomedical applications such as enzyme and cell immobilization and hydrogel formation. It is a dialdehyde with highly reactive aldehydic groups capable of forming covalent bonds with functional groups such as amines, thiols, phenols, hydroxyls, and imidazoles [246]. The optimal concentration of glutaraldehyde for chemical crosslinking is 1.0% (*v*/*v*), which preserves the cytocompatibility of composites [247]. GA is also employed as an activation reagent [14]. The GA stabilizes the composite by forming chemical bonds between the GA and the components [248].

A number of composite materials have been developed by crosslinking BC with gelatin and fibrin for tissue engineering. The amine groups of gelatin (or fibrin) and the hydroxyl groups of BC cellulose underwent a chemical reaction, forming hydrogen bonds [249].

A composite of BC and gelatin was synthesized for use as a small-diameter artificial blood vessel. After introducing fish gelatin into the fiber network of BC tubes, GA was used as a chemical crosslinking agent. The results demonstrated that the BC/gelatin composite tubes exhibited superior water permeability and nutrient transportation through the tube walls compared to BC. All BC/gelatin composite tubes could withstand pressure three times or higher than normal human blood pressure. In vitro cell culture experiments showed that human venous endothelial cells (HUVECs) and human smooth muscle cells seeded on the inner walls of BC/gelatin tubes had improved adhesion, proliferation, and differentiation potential, as well as anticoagulant and mechanical properties. Whole blood coagulation tests showed that BC/gelatin tubes outperformed BC tubes, with all samples exhibiting a hemolysis rate of less than 1.0%, meeting international medical device criteria [247].

Artificial blood vessels have been developed based on GA-treated BC/fibrin composites. The Young’s modulus and the time-dependent viscoelastic behavior of the composite were comparable to a reference small-diameter blood vessel. However, the strain at the break of the composite material (0.33) was still significantly lower than that of the native blood vessel (1.07) [250].

**Figure 10:**
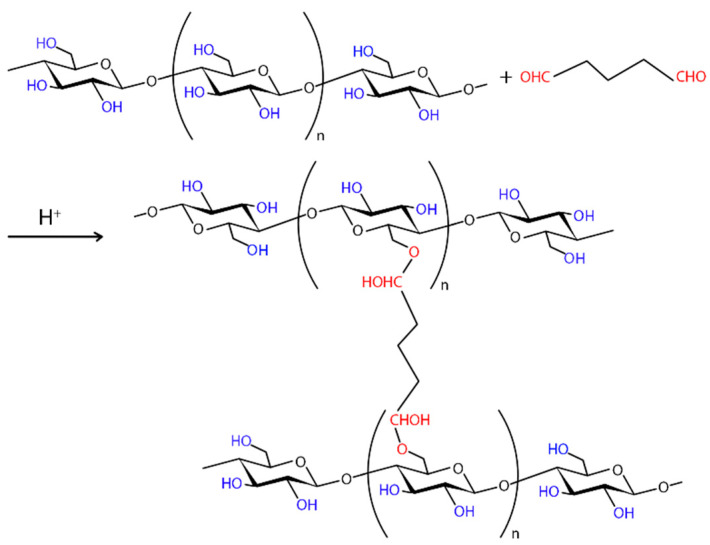
Schematic representation of the crosslinking reaction between glutaraldehyde and the hydroxyl groups of BC. The crosslinking of hydroxyl groups with GA is carried out in acidic conditions.

In certain studies, GA was utilized as a pre-activating agent for BC, facilitating the subsequent covalent immobilization of enzymes onto the activated BC (Figure 10).

The development of an in vitro enzymatic conversion process with GAD immobilized on the BC membrane was proposed for the efficient production of γ-Aminobutyric acid. Three different immobilization methods were compared. In the case of pre-activation BC with GA followed by adsorption, the enzyme was immobilized on BC via covalent binding and retained more than 96% of its original activity after seven cycles and had 89.17% of the activity of the native enzyme. After adsorption followed by crosslinking, the GAD activity on BC was 60.1% of the initial value and decreased rapidly over seven cycles. The membrane prepared by the physical adsorption method showed the highest GAD activity (95.11%), but the enzyme activity decreased exponentially with increasing cycle number of uses [45]. In another study using lipase as a model enzyme, the two-step immobilization strategy involving BC activation by GA and enzyme binding was also the most effective. The activity of the immobilized enzyme was 93.5% of that of the free enzyme. After six reactions, the activity was 76.7% (330 U) of that in the first reaction. Immobilized and free enzymes had identical activity at different pH and temperature levels. In addition, the immobilized lipase retained 60% of its initial activity after 15 cycles of use, in contrast to the free enzyme [14].

BCNC crosslinked by GA was used for the delivery of cyano-phycocyanin (C-PC). The C-PC was loaded onto the BCNC to make a carrier that keeps the C-PC stable, increases the time of drug release, and improves absorption in the gastrointestinal tract. The crosslinking process resulted in a BCNC structure with larger pores and enhanced stability. C-PC was immobilized on BCNC by physical adsorption, reaching 65.3% adsorption in 3 h. After 24 h, 69.46% of the C-PC was released from the BC, 47.39% from the BCNC, and 43.21% from the BCNC-GA [177].

- CDAP

The organic cyanating reagent 1-cyano-4-dimethylaminopyridinium tetrafluoroborate (CDAP) is used to activate polysaccharides, which could subsequently react with spacer reagents or directly with protein. In comparison to CNBr, CDAP is simpler to utilize, may be used at lower pH, has fewer negative effects and proteins can be directly linked to CDAP-activated polysaccharides [251,252]. Proteins interact with the cyanoester of CDAP-activated polysaccharides primarily through the unprotonated ε-amines of surface lysine residues, forming an isourea bond. It is possible that numerous multipoint connections may form between the polysaccharide and the protein, given that proteins have a large number of lysine residues that can react with the activated polysaccharide [253].

Acellular tissue-engineered vascular grafts have been developed based on BC. BCs were modified with heparin-chitosan, albumin, and fibronectin to encourage the growth of HUVECs or endothelial progenitor cells (EPCs). To prepare scaffolds with albumin or fibronectin, the BC surface was activated with CDAP. A portion of the BCs were coated with heparin by immersion in an EDC/NHS. BC-based grafts were characterized by good surgical controllability and burst pressure in a physiological environment. Fibronectin coating significantly promoted the adhesion and growth of VECs and EPCs, while albumin only promoted the adhesion of VECs, but the cells were functionally impaired. At the same time, fibronectin-modified surfaces were capable of capturing platelets via β1 and αIIbβ3 integrins, which can lead to increased thrombogenicity. Heparin-chitosan coating significantly improved EPC adhesion but not VEC adhesion [254].

- Citric acid

**Figure 11:**
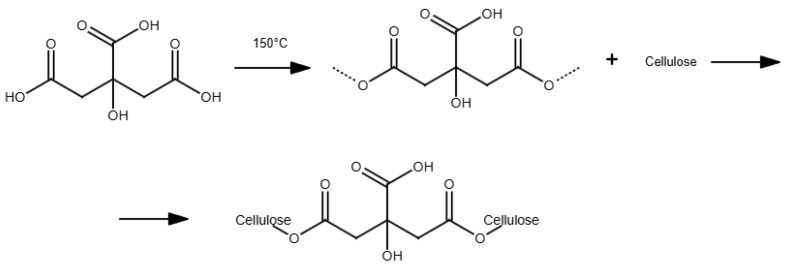
The crosslinking between BC and citric acid.

Citric acid (CA) is regarded as a safe and non-toxic crosslinker. CA has been employed to cross-link nanofibers and preserve the 3D structure of BC pellicles. One potential mechanism for the crosslinking process of BC with CA is the dehydration of BC as a result of the esterification process. This reaction produces an ester group that can bind to the accessible hydroxyl group of cellulose (Figure 11) [243].

The crosslinking of BC with CA and the functionalization of the resulting material with Ni- and Mg-ferrite magnetic nanoparticles were employed for the immobilization of lipase B from *Candida antarctica* and phospholipase A from *Aspergillus oryzae*. Both enzymes demonstrated significantly enhanced thermal stability at 60 °C, with a notable retention of residual activity at 70 °C, in contrast with the free form, which exhibited a notable decline. After 10 cycles of repeated use, the analyzed enzymes retained a notable amount of residual activity. However, there was a decline in the catalytic efficiency of phospholipase A and lipase after immobilization. In addition, there was a notable difference in immobilization efficiency between phospholipase A and lipase B [191].

- Physical crosslinking

Physical crosslinking has attracted much interest in hydrogel formation due to its ease of formulation and the fact that it does not require crosslinking chemicals [255]. This approach is primarily based on the generation of free radicals, which are then exposed to a high-energy source such as an electron beam, x-ray, or gamma ray [256,257].

**Figure 12:**
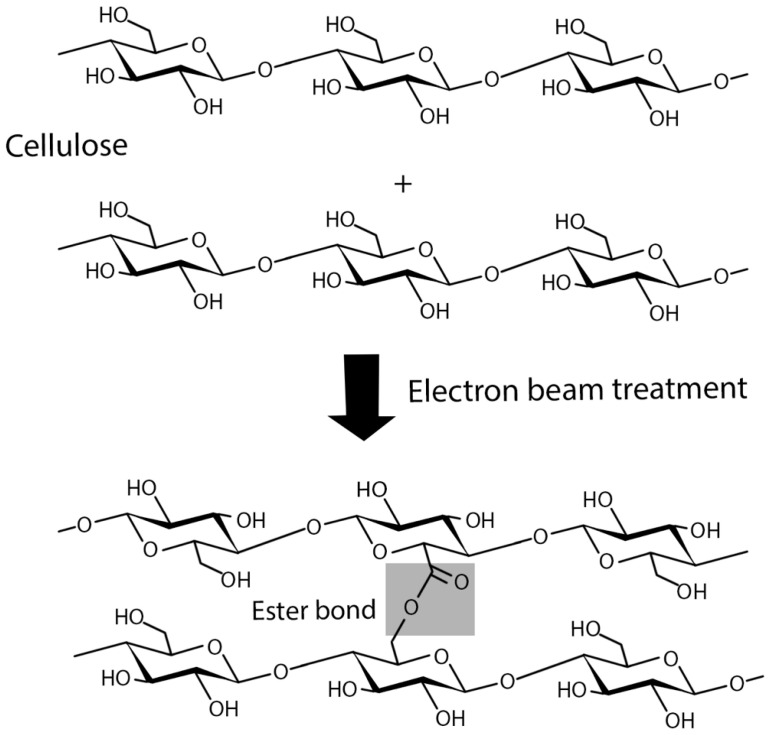
The effect of electron beam irradiation of cellulose.

Electron beam irradiation can be used to induce physical crosslinking (Figure 12), resulting in pure, sterile, and residue-free hydrogels. In addition, electron beam irradiation does not require the use of a radioactive source, reducing the possibility of harmful toxicity [258]. The electron beam creates patterns on BC’s surface without changing its chemical or crystalline structure [259]. BC membranes that have been irradiated are more swollen and biodegradable than non-irradiated ones [120]. These features make them excellent materials for use in drug delivery applications [106,260].

Stimuli-responsive BC-g-poly(acrylic acid) hydrogels were prepared by electron beam irradiation for the oral delivery of proteins. Bovine serum albumin (BSA) was a model protein. The cumulative release of BSA was less than 10% in simulated gastric fluid, demonstrating the ability of the hydrogels to protect BSA from the acidic environment of the stomach. Hydrogels had the maximum adhesiveness among the described hydrogels. The in vitro cytotoxicity test of the hydrogel demonstrated that the viability of Caco-2 cells was well above 90%. In addition, an ex vivo penetration study indicated that BSA penetration increased throughout the intestinal mucosal tissue, and acute oral toxicity tests demonstrated the safety of these hydrogels for in vivo applications [261].

#### 5.1.4. Modification of the Chemical Structure of Bacterial Cellulose

The hydroxyl groups of BC form both intra- and intermolecular hydrogen bonds, which limits the reactivity of the hydroxyl groups [262]. As a result, the hydroxyl groups cannot react directly with the functional groups present in proteins [199]. Furthermore, the loading of substances into BC by physical absorption does not result in sufficient interactions to enable long-term immobilization of protein molecules. The incorporation of functional groups [185] into the BC generates a carrier that can covalently immobilize biomolecules like proteins. For example, modification of its chemical structure of cellulose (derivatization) promotes the solubility in water of even hydrophobic derivatives [25].

The techniques for chemical modification of cellulose mostly include esterification, oxidation, etherification, carbamation, and amination, which are commonly carried out by creating reactive functional and charged groups on the surface by utilizing free hydroxyl groups [100,207]. Among the three hydroxyl groups, including two secondary hydroxyl groups and one primary hydroxyl group, the relative reactivity can generally be expressed in the following order: OH-C6 ≫ OH-C2 > OH-C3 [185,263]. Chemical coupling agents must be added to the main and secondary hydroxyl groups of the cellulose structure to immobilize proteins [44].

#### Oxidation

In order to regulate its degradability in vivo, BC can be selectively oxidized by oxidants, including TEMPO–NaClO–NaBr and NaIO_4_ [264]. The oxidized BC (OBC) membrane could act as a support matrix for the covalent immobilization of proteins [110]. For the improvement of the immobilization stability, the protein could be covalently conjugated to the OBC membrane via a Schiff base reaction [265]. It has been shown that oxidation affects the swelling and crystallinity of BC [106]. Furthermore, oxidation allows the optimization of BC degradability. Biodegradability through oxidation treatment and bioresorbability are aspects of BC that are being widely investigated for potential application in tissue engineering [266].

- Periodate

**Figure 13:**
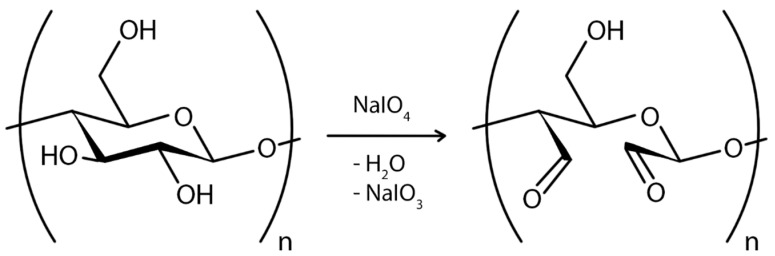
Schematic of sodium periodate oxidation of BC. This chemical process cleaves the chemical bonds between the C2 and C3 carbons of the D-glucopyranose ring, thereby allowing the insertion of two aldehyde groups per ring, resulting in the formation of two aldehyde groups per glucose unit and 2,3-dialdehyde cellulose (DAC).

Periodate ions (IO^4−^) can selectively oxidize secondary hydroxyl groups in cellulose (Figure 13) [267]. The aldehyde groups are useful for introducing various substituent groups such as carboxylic acids, hydroxyls, or imines [7]. In addition, the oxidation process reduces the negative charge density, increasing the possibility of intermolecular interactions with enzymes [7].

Porous 3D BC microspheres with collagen (COL) and bone morphogenetic protein 2 (BMP-2) were developed for bone tissue engineering. The resulting COL/BC/BMP-2 microspheres exhibited a porous structure with numerous interconnecting voids and a rough surface. These microspheres exhibited biocompatibility and enhanced the adhesion, proliferation, and differentiation of the mouse osteogenic cell line MC3T3-E1 cells. The adhesion rate of MC3T3-E1 cells to collagen was 86.7%, while the adhesion rate to BC was 66.7%. The mice MC3T3-E1 cells on COL/BC porous microspheres and COL/BC/BMP-2 microspheres produced more calcium nodules than the control [268].

Novel composite membranes created by Gorgieva et al. using BC membranes and gelatin biopolymers for tissue regeneration. The conjugation of OBC membranes with EDC significantly increased the physiological stability of gelatin. As a result, the resulting BC-gelatin membranes were found to have high swelling, degradation rate, and pH retention. MRC-5 cells adhered well to the porous gelatin regions of the resulting membrane, and it was not cytotoxic [139].

Double-modified BC (DMBC) was combined with soy protein isolate (SPI) to create a novel urethral scaffold. The number of adipose stem cells adhered to DMBC and DMBC/SPI material was significantly higher than that adhered to BC. The DMBC/SPI composite had low cytotoxicity but good biocompatibility on the third day of incubation with adipose stem cells. The in vivo results in New Zealand rabbits showed that DMBC/SPI did not induce an inflammatory response. Two weeks after surgery, inorganic tissue with a smooth urethral surface and continuous mucosa was produced in the DMBC/SPI group, while organic tissue was produced in the BC group. The DMBC/SPI acquired good degradation ability in vivo, while BC was not degradable in the rabbit body [143].

The DMBC was also created for the purpose of loading FGFR2-modified adipose-derived stem cells (ADSCs) for urethral repair. ADSCs are frequently employed in tissue repair due to their accessibility and capacity to release a variety of cytokines [269,270]. The secretory function and repair effects of ADSCs may be improved by targeted overexpression of FGFR2. The modification of BC involved oxidizing BC and followed sulfonation with NaHSO_3_. The lentiviral transfection with plasmid systems was used to create FGFR2-expressing ADSCs. The results demonstrated that the new composite with FGFR2-expressing ADSCs exhibited excellent repairability, with this ability correlated with angiogenesis. FGFR2 enhanced the osteogenic capacity of ADSCs without significantly affecting lipogenic capacity [271].

Vasconcelos et al. developed a bioactive dressing by immobilizing papain on oxidized BC (OBC) membranes. The OBC membrane was able to immobilize papain via covalent bonding (–C–NHR) and adsorption (ion exchange), with a recovered activity of 93.3%, an immobilization efficiency of 49.4%, and superior thermal properties OBC over BC. The release mechanisms of the BC–Papain and OBC–Papain membranes were anomalous and predominantly non-Fickian diffusion. The activities measured for wet oxidized BC and BC membranes showed no significant difference [110].

A spherical OBC was used as a carrier for the immobilization of industrial lipases. The stability and hydrolytic activity of lipase immobilized by covalent binding on spherical BC were significantly improved. Two optimal pH values (5 and 8) and a relatively low active temperature (30 °C) were achieved for optimal hydrolytic activity of lipases immobilized on BC, which was superior to free lipase (pH 9 and 40 °C) [184].

BC spheres also served as a carrier to immobilize the Lecitase^®^ Ultra (E.C.3.1.1.32, Sigma-Aldrich, St. Louis, MO, USA) enzyme. The OBC spheres were incubated with PEI and saturated with a mixture of Fe^2^+/Fe^3^+ ions. The obtained spheres OBC were then activated with 1% GA to immobilize the enzyme. The maximum yield for Lecitase^®^ Ultra immobilization was 70%. The immobilized enzyme exhibited had no significant impact on the enzyme’s K_M_ value. The immobilized enzyme retained more than 70% of its original activity after eight cycles of use and excellent storage stability, retaining 80% of its initial activity after four weeks at 4 °C [181].

Laccase and TiO_2_ were added to OBC to create a composite with the biocatalytic properties of laccase and the photocatalytic properties of TiO_2_. Immobilized laccase outperformed free laccase in terms of pH and temperature stability. The optimal pH for dye degradation was 5.0–6.0, while the optimal temperature was 40 °C. In addition, the immobilized laccase maintained a relative activity of 67% after ten cycles. Under UV irradiation, the oxidized BC/TiO_2_-Laccase composite degraded 95% of the dye within 3 h [186].

- TEMPO

**Figure 14:**
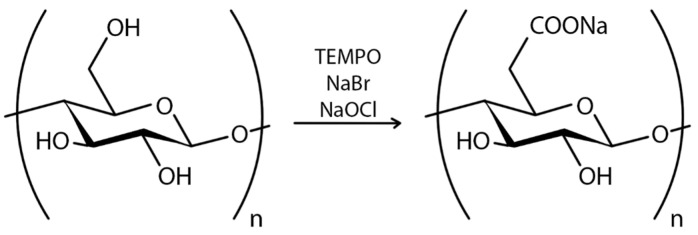
Schematic of TEMPO-mediated oxidation of BC. The process converts hydroxyl groups attached to a main carbon (C6) to aldehyde groups and ultimately to carboxylate, weakening hydrogen bonds and promoting mechanical degradation. The TEMPO-mediated oxidation process occurs at pH~10 and room temperature, with the use of NaClO, TEMPO, and NaBr.

One of the most effective pretreatments for BC is TEMPO oxidation, which selectively modifies the polymer under mild aqueous conditions (Figure 14). TEMPO oxidation creates carboxylate groups (–COO-Na^+^) that increase interchain electrostatic repulsion, leading to nanofibril disaggregation. BC membranes degraded by TEMPO oxidation have been investigated as stabilizers for food, topical, and pharmaceutical emulsions, replacing surfactants that often cause irritating reactions [272,273]. TEMPO-mediated oxidized BC scaffolds alone operate as potential tissue engineering scaffolds [274].

A bioadhesive composite based on the conjugation of involucrin antibody SY5 and BCNF has been developed. This system enables antigen–antibody interaction between SY5 on BCNF and involucrin exposed in the stratum corneum and epidermis. The antibodies covalently conjugated to oxidized BCNF using EDC/NHS. The BCNF–SY5 composite exhibited 2- to 3.5-fold higher adhesion than albumin-conjugated BCNF. BCNF-SY5 composite has been shown to effectively adhere to damaged skin and stimulate cell proliferation while preserving the intrinsic properties of the antibody [109].

The BC membranes oxidized by TEMPO have been utilized in the development of vaccines for aquatic animals. For this purpose, oxidized BC membranes were conjugated with ribavirin and NbE4 nanobody using EDC/NHS. The results of RT-qPCR analysis of the major capsid protein of LMBV demonstrated that the BC-ribavirin-NbE4 (BRN) therapy resulted in a notable reduction in virus abundance in infected largemouth bass. Furthermore, following the appropriate treatment, the BRN group exhibited reduced levels of inflammation-related factors. After seven days of treatment, the BRN group exhibited reduced expression levels of IRF-3 and IRF-6, indicating that IRF-3 and IRF-6 in the BRN group had returned to normal or pre-infectious levels [173].

#### Polymer Grafting on BC

Grafting is a process in which a parent polymer serves as the backbone, and branches of a second polymer are attached at various points. Polymer grafting increases the functional properties of the polymer by performing sulfonation, phosphorylation, carboxymethylation, and acetylation [275]. The most prevalent methods for BC modification include surface-induced atom transfer radical polymerization (ATRP), the conventional synthesis approach, and the crosslinking of silane coupling agents [174].

**Figure 15:**
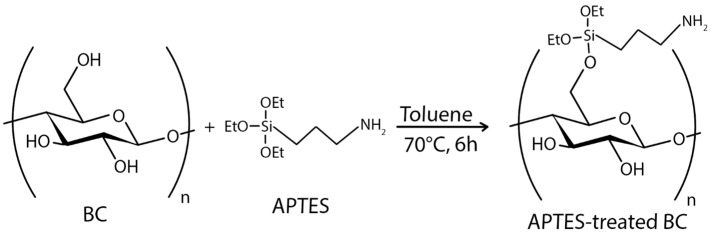
APTES treatment of BC. APTES has three functional reactive ethoxy groups and one amine group per silane molecule. APTES reacts with the hydroxyl groups (OH) on the BC surface, specifically, the OH groups attached to the C6 carbon of the glucose units in the cellulose. This reaction forms covalent bonds between the silane (Si–OH) groups of APTES and the cellulose OH groups.

Silanization of cellulose materials is a unique process that serves two distinct purposes. Firstly, it is used as an independent functionalization of cellulose. Secondly, it is employed as an intermediate step to introduce the necessary functionality for further modification. A highly efficient surface functionalization approach is the direct introduction of amino groups into BC using a silane coupling technique [185]. The silane agent most commonly used in this process is (aminopropyl)triethoxysilane (APTES) [276]. APTES is used to conduct the amination of BC (Figure 15). APTES does not alter the water absorption characteristics of BC [277]. It was found that the functionalization of the BC membrane with APTES introduces a new opportunity in click chemistry [278].

Ying et al. first used silanization of BC to immobilize HRP by binding GA. The amino-functionalized BC was activated with GA, and the HRP was covalently attached via its amino groups. The activity and reusability of the immobilized HRP were compared with those of the free enzyme. The optimal pH range for immobilized HRP (pH 5.5–8.5) was greater than that of the free enzyme (pH 6–8), and immobilized HRP was well adapted to ambient alkalinity. The relative activity of immobilized HRP was found to be higher by 90% than that of free HRP at temperatures 25–40 °C. Moreover, BC-immobilized HRP was reused effectively for 10 cycles, exhibiting greater than 70% of its original activity retention [185].

A BC scaffold functionalized with laminin and growth factors was prepared as a support structure for patterning and expansion of human embryonic stem (hES) cell-derived progenitor cells. Dopaminergic ventral midbrain (VM) progenitor cells are being used in cell replacement therapies for Parkinson’s disease. The BC was modified using the silanization method, and laminin and the growth factors BDNF and GDNF were immobilized on the BC surface via a covalent bond. The functionalization of BC resulted in an improved differentiation rate of cells after plating on BC. The viability of hES-derived VM progenitor cells seeded on different substrates was not affected by cellulose functionalization, regardless of whether BC had been modified with laminin + BDNF + GDNF or laminin alone. Furthermore, the expression of early dopaminergic markers in these cells was enhanced by the growth factor functionalization of BC. The modification of BC with growth factors prevents protein leakage while also providing cells with a long-term supply of growth factors required for proper differentiation and development of VM progenitor cells [172].

An active, non-resorbable guided tissue regeneration membrane by conjugating BC with recombinant human osteopontin (OPN) was proposed by Klinthoopthamrong et al. Surface-initiated reversible addition-fragmentation chain transfer (RAFT) polymerization was used to graft PAA onto the surface of BC. Then, p-rhOPN or rhOPN (commercial preparation) was conjugated to the PAA-grafted BC. Conjugated p-rhOPN has an immobilization efficiency of 97%. Both p-rhOPN-BC and rhOPN-BC demonstrated enhanced capabilities in promoting human periodontal ligament stem cell adhesion, osteogenic differentiation, and calcium deposition levels when compared to BC alone [15].

**Figure 16:**
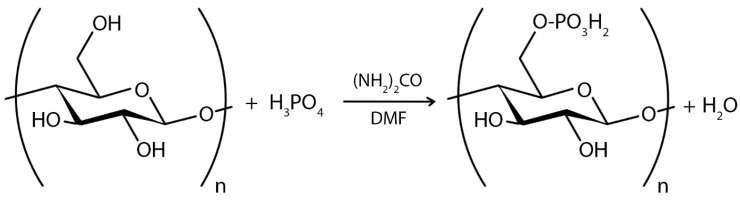
Schematic of phosphorylation of BC for immobilization of proteins. The phosphorylation process involves the substitution of the C-6 primary hydroxyl group with phosphoric acid groups.

The addition of phosphate moieties to the cellulose backbone represents a significant approach for the preparation of a diverse array of phosphate derivatives from cellulosic materials. Furthermore, phosphate-ester functionalized cellulosic materials are compatible with calcium phosphate, thereby enabling the formation of novel hybrid materials that can be utilized in bone tissue engineering and drug delivery applications. It has been widely reported that concentrated H_3_PO_4_ has been used extensively as an effective phosphating agent (Figure 16) [279].

Phosphorylated BC (PBC) was found to be an attractive adsorbent with a large adsorption capacity for proteins [280]. In the investigation of the adsorption of proteins on obtained PBC, it was discovered that it has a much larger specific surface area than phosphorylated plant cellulose (PPC). The adsorption capacity for the protein increased as the percentage of phosphorylation increased. The adsorption capacity of PBC was much higher than that of PPC, even though their phosphorylation percentages were similar [280].

### 5.2. In Situ Bacterial Cellulose Modification

A variety of materials, including polyaniline [281], collagen [282], hyaluronan [155,166], xanthan gum [283], CMC [284,285,286], and sodium alginate [287,288] have been already utilized to modify BC in situ. These modifications were intended to enhance the morphological and physicochemical properties of BCs for biomedical applications [202]. To date, several modifications of BC in situ have been performed for protein immobilization in tissue engineering. The application of proteins as functional molecules to modify BC during microbial fermentation, as an alternative to conventional chemical techniques, has the potential to result in a more sustainable process. The in situ-modified materials are biocompatible and can undergo decay in a regulated manner [289].

A collagen-BC composite was prepared by incorporating collagen into the incubation medium of *A. xylinum*. The collagen–BC composite exhibited a well-interconnected porous network structure and a large surface area required for cell attachment and vascularization. The crystal structure of BC also underwent a transformation when collagen was introduced into the incubation medium of *A. xylinum* [282].

A novel approach for 3D cell culture of tumor cells has recently been developed using BC. The BC was modified in situ with hyaluronic acid and gelatin to create a bioengineered tumor model containing a network of nanofibers and a human glioblastoma cell line (U251). Their findings indicated that the BC/hyaluronic acid/gelatin composite scaffold exhibited moderately hydrophilic properties, influencing cell adhesion and proliferation behavior significantly. The U251 cells exhibited normal morphology and good adhesion and demonstrated excellent viability, forming multilayered and compact cell clusters [155].

In situ modification of BC with CMC and conjugation with anti-HSA affibody was performed to use BC as a matrix for selective biofiltration of blood proteins. CMC–BC composites with conjugated anti-HSA affibodies demonstrated superior binding efficacy for HSA compared to TEMPO-oxidized BC composites. The carboxylated cellulose conjugated with anti-HSA via EDC/NHS exhibited approximately eight-fold higher HSA-specific binding capacity than the carboxylated cellulose surface with physically adsorbed anti-HSA. Affibody conjugation increased the affinity and specificity of CMC–BC tubes to capture target molecules, and the presence of CMC in the BC network reduced irreversible structural changes during the drying process [286].

The protein BslA (*B. subtilis* biofilm protein, bacterial hydrophobin) was employed for the modification of BC in a localized manner [289]. BslA has the capacity to form a hydrophobic film that coats the biofilm surface, rendering it water-repellent [290]. The results of in situ modification demonstrate that BslA has the potential to cause structural and mechanical changes to the BC fiber network, thereby creating a stronger, less brittle material with enhanced potential for use in a wide range of applications. However, higher concentrations of BslA and BslA–CBM have been observed to delay the formation of the BC pellicle [289].

## 6. Conclusions

BC has recently emerged as one of the most popular biomaterials among engineers and scientists [82]. BC is a promising flexible material with a high water-holding capacity, outstanding mechanical strength, and a low cost [24], making it an ideal solution for immobilizing a wide range of molecules, nanoparticles, and cells. Due to its unique mechanical properties, its renewable, bio-based, biodegradable properties [53], and its lack of contaminants [214], there has been considerable effort to incorporate BC into a variety of commercial products. A nontoxic, biocompatible wound dressing (Cellulose Solutions LLC), antimicrobial (Axcelon Biopolymers, JeNacell), and other commercial BC-based dressings (Biofill^®^, Cellulon^®^, and Gengiflex^®^) have been developed recently [25]. In addition, Kusano Sakko Inc. (KSI) has reported utilizing BC as a matrix for the delivery of anti-cancer medications with controlled drug release [50].

BC is a material that is employed in a multitude of biomedical developments due to its capacity to assume novel properties through the process of modification [291]. Recently, it has been demonstrated that the incorporation of BCNF into fish myofibrillar protein substrate can impart beneficial effects on the physical properties of the resulting films, leading to an enhancement in water resistance compared to the control film [292]. Due to its similarity to human tissues, BC has great potential for application in regenerative therapy, organ replacement, targeting the capability of drug delivery systems, and immobilizing proteins [62]. Immobilization of proteins on BC is one method to improve protein properties [293] and protection of their 3D structure and activity [49,294]. Currently, the majority of published articles on protein immobilization on BC are devoted to the creation of wound healing, tissue scaffolds, and the immobilization of enzymes. The BC has been compounded with proteins for applications such as enhancing osteoblast cell growth in bone regeneration, guiding fibroblast/endothelial cells in wound healing [295], blood vessel replacement, composite with antibacterial properties, to trap tumor cells, for developing attractive adsorbent, improving enzyme stability, etc. [25]. Several studies have indicated that BC can be used as a delivery system for both protein and non-protein drugs [293]. Immobilization could not only increase the activity of enzymes but also allow them to be reused [296].

Despite its remarkable potential, BC is currently too expensive to produce on a large scale. As a result, it is not commonly used as a replacement for plant cellulose [297]. The high production cost of BC due to expensive intermediate components, as well as the low yield and productivity, are significant barriers to the commercialization of BC-based products. The issue of high production cost due to the use of expensive intermediate components has been addressed to some extent by exploring the possibility of various agro-industrial wastes and low-cost substrates [298,299]. In addition, the use of low-cost agro-industrial waste products such as corn steep liquor in biomass production can not only reduce costs but also minimize environmental impact [300]. While biomaterials typically result in reduced greenhouse gas emissions compared to conventional materials, they may potentially contribute to increased eutrophication and stratospheric ozone depletion [301].

The yield and productivity of BC have been significantly increased through the development of modern reactors, the investigation of efficient bacterial strains, and the generation of novel strains [128,297,302,303]. Furthermore, co-cultivation represents an effective approach to enhancing BC production and obtaining modified BC pellicles [304]. It has been demonstrated that BC generated through the co-cultivation of *Aureobasidium pullulans* and BC producer *G. hansenii* exhibits 4.5–6 times greater elasticity and a 22.4% increase in BC production compared to that of BC produced through other methods [305].

Another serious problem is that unmodified BC does not have antibacterial, antioxidant, anticancer, or electromagnetic properties [306] and has low biocompatibility due to the lack of attachment sites. The physical stability and relatively low degradation rate of cellulose in the human body may pose challenges for BC in certain biomedical applications. Therefore, several strategies have been proposed to improve the biocompatibility of BC, including surface modification, porosity modification, and preparation of BC composites [307]. BC can be functionalized with polymers, nanomaterials, antibiotics, peptides, etc. [299,302,308]. A major focus of current BC modification research has been the development of cheaper and more environmentally friendly methods. Despite many years of research on BC modification, there is still much to be discovered in biological applications [174].

## Supplementary Materials

The following supporting information can be downloaded at https://www.mdpi.com/article/10.3390/polym16172468/s1, Table S1: Summary of BC modifications for protein immobilization in biomedicine.

## References

[1] Kharisov B.I., Kharissova O.V., Ortiz-Mendez U. (2016). Drug Delivery: LyoCell® Technology—A Lipidic Drug Delivery System Based on Reverse Cubic and Hexagonal Phase Lyotropic Liquid Crystalline Nanoparticles. CRC Concise Encyclopedia of Nanotechnology.

[2] Maghraby Y.R., El-Shabasy R.M., Ibrahim A.H., Azzazy H.M.E.-S. (2023). Enzyme Immobilization Technologies and Industrial Applications. ACS Omega.

[3] Liang J.F., Li Y.T., Yang V.C. (2000). Biomedical Application of Immobilized Enzymes. J. Pharm. Sci..

[4] Pinmanee P., Sompinit K., Jantimaporn A., Khongkow M., Haltrich D., Nimchua T., Sukyai P. (2023). Purification and Immobilization of Superoxide Dismutase Obtained from Saccharomyces Cerevisiae TBRC657 on Bacterial Cellulose and Its Protective Effect against Oxidative Damage in Fibroblasts. Biomolecules.

[5] Jesionowski T., Zdarta J., Krajewska B. (2014). Enzyme Immobilization by Adsorption: A Review. Adsorption.

[6] Klimek K., Ginalska G. (2020). Proteins and Peptides as Important Modifiers of the Polymer Scaffolds for Tissue Engineering Applications—A Review. Polymers.

[7] Isobe N., Lee D.-S., Kwon Y.-J., Kimura S., Kuga S., Wada M., Kim U.-J. (2011). Immobilization of Protein on Cellulose Hydrogel. Cellulose.

[8] Lyu X., Gonzalez R., Horton A., Li T. (2021). Immobilization of Enzymes by Polymeric Materials. Catalysts.

[9] Tang S., Chi K., Xu H., Yong Q., Yang J., Catchmark J.M. (2021). A Covalently Cross-Linked Hyaluronic Acid/Bacterial Cellulose Composite Hydrogel for Potential Biological Applications. Carbohydr. Polym..

[10] Liu Y., Chen J.Y. (2016). Enzyme Immobilization on Cellulose Matrixes. J. Bioact. Compat. Polym..

[11] Ul-Islam M., Khan T., Park J.K. (2012). Nanoreinforced Bacterial Cellulose–Montmorillonite Composites for Biomedical Applications. Carbohydr. Polym..

[12] Shah N., Ul-Islam M., Khattak W.A., Park J.K. (2013). Overview of Bacterial Cellulose Composites: A Multipurpose Advanced Material. Carbohydr. Polym..

[13] He W., Xu J., Zheng Y., Chen J., Yin Y., Mosselhy D.A., Zou F., Ma M., Liu X. (2022). Bacterial Cellulose/Soybean Protein Isolate Composites with Promoted Inflammation Inhibition, Angiogenesis and Hair Follicle Regeneration for Wound Healing. Int. J. Biol. Macromol..

[14] Wu S., Wu S., Su F. (2017). Novel Process for Immobilizing an Enzyme on a Bacterial Cellulose Membrane through Repeated Absorption. J. Chem. Technol. Biotechnol..

[15] Klinthoopthamrong N., Chaikiawkeaw D., Phoolcharoen W., Rattanapisit K., Kaewpungsup P., Pavasant P., Hoven V.P. (2020). Bacterial Cellulose Membrane Conjugated with Plant-Derived Osteopontin: Preparation and Its Potential for Bone Tissue Regeneration. Int. J. Biol. Macromol..

[16] Qian L., Yang Y., Xu T., Zhang S., Nica V., Tang R., Song W. (2022). Fabrication of Efficient Protein Imprinted Materials Based on Pearl Necklace-like MOFs Bacterial Cellulose Composites. Carbohydr. Polym..

[17] Fu L., Zhang J., Yang G. (2013). Present Status and Applications of Bacterial Cellulose-Based Materials for Skin Tissue Repair. Carbohydr. Polym..

[18] Ullah M.W., Manan S., Kiprono S.J., Ul-Islam M., Yang G. (2019). Synthesis, Structure, and Properties of Bacterial Cellulose. Nanocellulose.

[19] Ullah M.W., Ul-Islam M., Khan S., Kim Y., Park J.K. (2015). Innovative Production of Bio-Cellulose Using a Cell-Free System Derived from a Single Cell Line. Carbohydr. Polym..

[20] Silva I.G.R.d., Pantoja B.T.d.S., Almeida G.H.D.R., Carreira A.C.O., Miglino M.A. (2022). Bacterial Cellulose and ECM Hydrogels: An Innovative Approach for Cardiovascular Regenerative Medicine. Int. J. Mol. Sci..

[21] Helenius G., Bäckdahl H., Bodin A., Nannmark U., Gatenholm P., Risberg B. (2006). In Vivo Biocompatibility of Bacterial Cellulose. J. Biomed. Mater. Res. Part A.

[22] Arikibe J.E., Lata R., Kuboyama K., Ougizawa T., Rohindra D. (2019). PH-Responsive Studies of Bacterial Cellulose /Chitosan Hydrogels Crosslinked with Genipin: Swelling and Drug Release Behaviour. ChemistrySelect.

[23] Lupașcu R.E., Ghica M.V., Dinu-Pîrvu C.-E., Popa L., Velescu B.Ș., Arsene A.L. (2022). An Overview Regarding Microbial Aspects of Production and Applications of Bacterial Cellulose. Materials.

[24] Choi S.M., Rao K.M., Zo S.M., Shin E.J., Han S.S. (2022). Bacterial Cellulose and Its Applications. Polymers.

[25] Gregory D.A., Tripathi L., Fricker A.T.R., Asare E., Orlando I., Raghavendran V., Roy I. (2021). Bacterial Cellulose: A Smart Biomaterial with Diverse Applications. Mater. Sci. Eng. R Rep..

[26] Gao M., Li J., Bao Z., Hu M., Nian R., Feng D., An D., Li X., Xian M., Zhang H. (2019). A Natural in Situ Fabrication Method of Functional Bacterial Cellulose Using a Microorganism. Nat. Commun..

[27] Saleh A.K., El-Gendi H., El-Fakharany E.M., Owda M.E., Awad M.A., Kamoun E.A. (2022). Exploitation of Cantaloupe Peels for Bacterial Cellulose Production and Functionalization with Green Synthesized Copper Oxide Nanoparticles for Diverse Biological Applications. Sci. Rep..

[28] Farooq U., Ullah M.W., Yang Q., Aziz A., Xu J., Zhou L., Wang S. (2020). High-Density Phage Particles Immobilization in Surface-Modified Bacterial Cellulose for Ultra-Sensitive and Selective Electrochemical Detection of Staphylococcus Aureus. Biosens. Bioelectron..

[29] Jayani T., Sanjeev B., Marimuthu S., Uthandi S. (2020). Bacterial Cellulose Nano Fiber (BCNF) as Carrier Support for the Immobilization of Probiotic, Lactobacillus Acidophilus 016. Carbohydr. Polym..

[30] Żywicka A., Banach A., Junka A.F., Drozd R., Fijałkowski K. (2019). Bacterial Cellulose as a Support for Yeast Immobilization—Correlation between Carrier Properties and Process Efficiency. J. Biotechnol..

[31] Swingler S., Gupta A., Gibson H., Kowalczuk M., Heaselgrave W., Radecka I. (2021). Recent Advances and Applications of Bacterial Cellulose in Biomedicine. Polymers.

[32] Chung C.K., Beekmann U., Kralisch D., Bierau K., Chan A., Ossendorp F., Cruz L.J. (2022). Bacterial Cellulose as Drug Delivery System for Optimizing Release of Immune Checkpoint Blocking Antibodies. Pharmaceutics.

[33] Pavaloiu R.-D., Stoica A., Stroescu M., Dobre T. (2014). Controlled Release of Amoxicillin from Bacterial Cellulose Membranes. Open Chem..

[34] Shao W., Liu H., Wang S., Wu J., Huang M., Min H., Liu X. (2016). Controlled Release and Antibacterial Activity of Tetracycline Hydrochloride-Loaded Bacterial Cellulose Composite Membranes. Carbohydr. Polym..

[35] Cacicedo M.L., Islan G.A., Drachemberg M.F., Alvarez V.A., Bartel L.C., Bolzán A.D., Castro G.R. (2018). Hybrid Bacterial Cellulose–Pectin Films for Delivery of Bioactive Molecules. New J. Chem..

[36] Cacicedo M.L., Pacheco G., Islan G.A., Alvarez V.A., Barud H.S., Castro G.R. (2020). Chitosan-Bacterial Cellulose Patch of Ciprofloxacin for Wound Dressing: Preparation and Characterization Studies. Int. J. Biol. Macromol..

[37] Junka A., Bartoszewicz M., Dziadas M., Szymczyk P., Dydak K., Żywicka A., Owczarek A., Bil-Lula I., Czajkowska J., Fijałkowski K. (2020). Application of Bacterial Cellulose Experimental Dressings Saturated with Gentamycin for Management of Bone Biofilm in Vitro and Ex Vivo. J. Biomed. Mater. Res. Part B Appl. Biomater..

[38] Trovatti E., Freire C.S.R., Pinto P.C., Almeida I.F., Costa P., Silvestre A.J.D., Neto C.P., Rosado C. (2012). Bacterial Cellulose Membranes Applied in Topical and Transdermal Delivery of Lidocaine Hydrochloride and Ibuprofen: In Vitro Diffusion Studies. Int. J. Pharm..

[39] Cacicedo M.L., León I.E., Gonzalez J.S., Porto L.M., Alvarez V.A., Castro G.R. (2016). Modified Bacterial Cellulose Scaffolds for Localized Doxorubicin Release in Human Colorectal HT-29 Cells. Colloids Surf. B Biointerfaces.

[40] Subtaweesin C., Woraharn W., Taokaew S., Chiaoprakobkij N., Sereemaspun A., Phisalaphong M. (2018). Characteristics of Curcumin-Loaded Bacterial Cellulose Films and Anticancer Properties against Malignant Melanoma Skin Cancer Cells. Appl. Sci..

[41] Akagi S., Ando H., Fujita K., Shimizu T., Ishima Y., Tajima K., Matsushima T., Kusano T., Ishida T. (2021). Therapeutic Efficacy of a Paclitaxel-Loaded Nanofibrillated Bacterial Cellulose (PTX/NFBC) Formulation in a Peritoneally Disseminated Gastric Cancer Xenograft Model. Int. J. Biol. Macromol..

[42] Hamimed S., Abdeljelil N., Landoulsi A., Chatti A., Aljabali A.A.A., Barhoum A. (2022). Bacterial Cellulose Nanofibers. Handbook of Nanocelluloses.

[43] Wu S.-C., Lia Y.-K. (2008). Application of Bacterial Cellulose Pellets in Enzyme Immobilization. J. Mol. Catal. B Enzym..

[44] Vasconcelos N.F., Andrade F.K., Vieira L.d.A.P., Vieira R.S., Vaz J.M., Chevallier P., Mantovani D., Borges M.d.F., Rosa M. (2020). de F. Oxidized Bacterial Cellulose Membrane as Support for Enzyme Immobilization: Properties and Morphological Features. Cellulose.

[45] Yao W., Wu X., Zhu J., Sun B., Miller C. (2013). In Vitro Enzymatic Conversion of γ-Aminobutyric Acid Immobilization of Glutamate Decarboxylase with Bacterial Cellulose Membrane (BCM) and Non-Linear Model Establishment. Enzym. Microb. Technol..

[46] Bacterial Cellulose Market, by Product Type (Industrial Grade and Technical Grade), by Application (Composites Materials, Nonwovens Absorbent Webs, Paper and Board, and Food Products), by Method, by End-Use Industry, and by Region Forecast to 2032. https://www.emergenresearch.com/industry-report/bacterial-cellulose-market.

[47] Bacterial Cellulose Market Analysis & Forecast 2024–2030. https://www.profsharemarketresearch.com/bacterial-cellulose-market-report/.

[48] Jadczak K., Ochędzan-Siodłak W. (2023). Bacterial Cellulose: Biopolymer with Novel Medical Applications. J. Biomater. Appl..

[49] Pötzinger Y., Kralisch D., Fischer D. (2017). Bacterial Nanocellulose: The Future of Controlled Drug Delivery?. Ther. Deliv..

[50] Ho Y.-S., Fahad Halim A.F.M., Islam M.T. (2022). The Trend of Bacterial Nanocellulose Research Published in the Science Citation Index Expanded From 2005 to 2020: A Bibliometric Analysis. Front. Bioeng. Biotechnol..

[51] Moniri M., Boroumand Moghaddam A., Azizi S., Abdul Rahim R., Bin Ariff A., Zuhainis Saad W., Navaderi M., Mohamad R. (2017). Production and Status of Bacterial Cellulose in Biomedical Engineering. Nanomaterials.

[52] Adachi O., Moonmangmee D., Toyama H., Yamada M., Shinagawa E., Matsushita K. (2003). New Developments in Oxidative Fermentation. Appl. Microbiol. Biotechnol..

[53] Vadanan S.V., Basu A., Lim S. (2022). Bacterial Cellulose Production, Functionalization, and Development of Hybrid Materials Using Synthetic Biology. Polym. J..

[54] Prilepskii A., Nikolaev V., Klaving A. (2023). Conductive Bacterial Cellulose: From Drug Delivery to Flexible Electronics. Carbohydr. Polym..

[55] Fernandes I.d.A.A., Pedro A.C., Ribeiro V.R., Bortolini D.G., Ozaki M.S.C., Maciel G.M., Haminiuk C.W.I. (2020). Bacterial Cellulose: From Production Optimization to New Applications. Int. J. Biol. Macromol..

[56] Fontana J.D., Koop H.S., Tiboni M., Grzybowski A., Pereira A., Kruger C.D., da Silva M.G.R., Wielewski L.P. (2017). New Insights on Bacterial Cellulose. Food Biosynthesis.

[57] Feng X., Ge Z., Wang Y., Xia X., Zhao B., Dong M. (2024). Production and Characterization of Bacterial Cellulose from Kombucha-Fermented Soy Whey. Food Prod. Process. Nutr..

[58] Tang W., Jia S., Jia Y., Yang H. (2010). The Influence of Fermentation Conditions and Post-Treatment Methods on Porosity of Bacterial Cellulose Membrane. World J. Microbiol. Biotechnol..

[59] Bäckdahl H., Esguerra M., Delbro D., Risberg B., Gatenholm P. (2008). Engineering Microporosity in Bacterial Cellulose Scaffolds. J. Tissue Eng. Regen. Med..

[60] Tang K.Y., Heng J.Z.X., Chai C.H.T., Chan C.Y., Low B.Q.L., Chong S.M.E., Loh H.Y., Li Z., Ye E., Loh X.J. (2022). Modified Bacterial Cellulose for Biomedical Applications. Chem. Asian J..

[61] Zhang L.-K., Du S., Wang X., Jiao Y., Yin L., Zhang Y., Guan Y.-Q. (2019). Bacterial Cellulose Based Composites Enhanced Transdermal Drug Targeting for Breast Cancer Treatment. Chem. Eng. J..

[62] Hu W., Chen S., Yang J., Li Z., Wang H. (2014). Functionalized Bacterial Cellulose Derivatives and Nanocomposites. Carbohydr. Polym..

[63] Ul-Islam M., Khan S., Fatima A., Ahmad M.W., Khan M.S., Ul Islam S., Manan S., Ullah M.W. (2022). Production of Bio-Cellulose from Renewable Resources: Properties and Applications. Renewable Polymers and Polymer-Metal Oxide Composites.

[64] Ciolacu D.E., Nicu R., Ciolacu F. (2020). Cellulose-Based Hydrogels as Sustained Drug-Delivery Systems. Materials.

[65] Jankau J., Błażyńska-Spychalska A., Kubiak K., Jędrzejczak-Krzepkowska M., Pankiewicz T., Ludwicka K., Dettlaff A., Pęksa R. (2022). Bacterial Cellulose Properties Fulfilling Requirements for a Biomaterial of Choice in Reconstructive Surgery and Wound Healing. Front. Bioeng. Biotechnol..

[66] Klemm D., Cranston E.D., Fischer D., Gama M., Kedzior S.A., Kralisch D., Kramer F., Kondo T., Lindström T., Nietzsche S. (2018). Nanocellulose as a Natural Source for Groundbreaking Applications in Materials Science: Today’s State. Mater. Today.

[67] Basu A., Strømme M., Ferraz N. (2018). Towards Tunable Protein-Carrier Wound Dressings Based on Nanocellulose Hydrogels Crosslinked with Calcium Ions. Nanomaterials.

[68] Huo Y., Liu Y., Xia M., Du H., Lin Z., Li B., Liu H. (2022). Nanocellulose-Based Composite Materials Used in Drug Delivery Systems. Polymers.

[69] Monika P., Chandraprabha M.N., Rangarajan A., Waiker P.V., Chidambara Murthy K.N. (2022). Challenges in Healing Wound: Role of Complementary and Alternative Medicine. Front. Nutr..

[70] Lin Y.-K., Chen K.-H., Ou K.-L., Liu M. (2011). Effects of Different Extracellular Matrices and Growth Factor Immobilization on Biodegradability and Biocompatibility of Macroporous Bacterial Cellulose. J. Bioact. Compat. Polym..

[71] Zhang W., Liu Y., Zhang H. (2021). Extracellular Matrix: An Important Regulator of Cell Functions and Skeletal Muscle Development. Cell Biosci..

[72] Sharda D., Kaur P., Choudhury D. (2023). Protein-Modified Nanomaterials: Emerging Trends in Skin Wound Healing. Discov. Nano.

[73] Ben Ayed H., Bardaa S., Moalla D., Jridi M., Maalej H., Sahnoun Z., Rebai T., Jacques P., Nasri M., Hmidet N. (2015). Wound Healing and in Vitro Antioxidant Activities of Lipopeptides Mixture Produced by Bacillus Mojavensis A21. Process Biochem..

[74] Savitskaya I.S., Shokatayeva D.H., Kistaubayeva A.S., Ignatova L.V., Digel I.E. (2019). Antimicrobial and Wound Healing Properties of a Bacterial Cellulose Based Material Containing B. Subtilis Cells. Heliyon.

[75] Tudoroiu E.-E., Dinu-Pîrvu C.-E., Albu Kaya M.G., Popa L., Anuța V., Prisada R.M., Ghica M.V. (2021). An Overview of Cellulose Derivatives-Based Dressings for Wound-Healing Management. Pharmaceuticals.

[76] Zhong S.P., Zhang Y.Z., Lim C.T. (2010). Tissue Scaffolds for Skin Wound Healing and Dermal Reconstruction. WIREs Nanomed. Nanobiotechnol..

[77] Li M., Xia W., Khoong Y.M., Huang L., Huang X., Liang H., Zhao Y., Mao J., Yu H., Zan T. (2023). Smart and Versatile Biomaterials for Cutaneous Wound Healing. Biomater. Res..

[78] Orlando I., Basnett P., Nigmatullin R., Wang W., Knowles J.C., Roy I. (2020). Chemical Modification of Bacterial Cellulose for the Development of an Antibacterial Wound Dressing. Front. Bioeng. Biotechnol..

[79] Portela R., Leal C.R., Almeida P.L., Sobral R.G. (2019). Bacterial Cellulose: A Versatile Biopolymer for Wound Dressing Applications. Microb. Biotechnol..

[80] Lin S.-P., Loira Calvar I., Catchmark J.M., Liu J.-R., Demirci A., Cheng K.-C. (2013). Biosynthesis, Production and Applications of Bacterial Cellulose. Cellulose.

[81] de Amorim J.D.P., da Silva Junior C.J.G., de Medeiros A.D.M., do Nascimento H.A., Sarubbo M., de Medeiros T.P.M., Costa A.F.d.S., Sarubbo L.A. (2022). Bacterial Cellulose as a Versatile Biomaterial for Wound Dressing Application. Molecules.

[82] Raut M., Asare E., Syed Mohamed S., Amadi E., Roy I. (2023). Bacterial Cellulose-Based Blends and Composites: Versatile Biomaterials for Tissue Engineering Applications. Int. J. Mol. Sci..

[83] He W., Wu J., Xu J., Mosselhy D.A., Zheng Y., Yang S. (2021). Bacterial Cellulose: Functional Modification and Wound Healing Applications. Adv. Wound Care.

[84] Kavitha K.V. (2014). Choice of Wound Care in Diabetic Foot Ulcer: A Practical Approach. World J. Diabetes.

[85] Abdelrahman T., Newton H. (2011). Wound Dressings: Principles and Practice. Surgery.

[86] Meftahi A., Khajavi R., Rashidi A., Sattari M., Yazdanshenas M.E., Torabi M. (2010). The Effects of Cotton Gauze Coating with Microbial Cellulose. Cellulose.

[87] Wiegand C., Moritz S., Hessler N., Kralisch D., Wesarg F., Müller F.A., Fischer D., Hipler U.-C. (2015). Antimicrobial Functionalization of Bacterial Nanocellulose by Loading with Polihexanide and Povidone-Iodine. J. Mater. Sci. Mater. Med..

[88] Sosnik A., Seremeta K. (2017). Polymeric Hydrogels as Technology Platform for Drug Delivery Applications. Gels.

[89] Li Y., Wang S., Huang R., Huang Z., Hu B., Zheng W., Yang G., Jiang X. (2015). Evaluation of the Effect of the Structure of Bacterial Cellulose on Full Thickness Skin Wound Repair on a Microfluidic Chip. Biomacromolecules.

[90] Moradpoor H., Mohammadi H., Safaei M., Mozaffari H.R., Sharifi R., Gorji P., Sulong A.B., Muhamad N., Ebadi M. (2022). Recent Advances on Bacterial Cellulose-Based Wound Management: Promises and Challenges. Int. J. Polym. Sci..

[91] Meng S., Wu H., Xiao D., Lan S., Dong A. (2023). Recent Advances in Bacterial Cellulose-Based Antibacterial Composites for Infected Wound Therapy. Carbohydr. Polym..

[92] Jabbari F., Babaeipour V. (2024). Bacterial Cellulose as an Ideal Potential Treatment for Burn Wounds: A Comprehensive Review. Wound Repair Regen..

[93] Rajwade J.M., Paknikar K.M., Kumbhar J.V. (2015). Applications of Bacterial Cellulose and Its Composites in Biomedicine. Appl. Microbiol. Biotechnol..

[94] Taokaew S., Phisalaphong M., Newby B.Z. (2015). Modification of Bacterial Cellulose with Organosilanes to Improve Attachment and Spreading of Human Fibroblasts. Cellulose.

[95] Czaplewski L., Bax R., Clokie M., Dawson M., Fairhead H., Fischetti V.A., Foster S., Gilmore B.F., Hancock R.E.W., Harper D. (2016). Alternatives to Antibiotics—A Pipeline Portfolio Review. Lancet Infect. Dis..

[96] Sulaeva I., Henniges U., Rosenau T., Potthast A. (2015). Bacterial Cellulose as a Material for Wound Treatment: Properties and Modifications. A Review. Biotechnol. Adv..

[97] Zheng L., Li S., Luo J., Wang X. (2020). Latest Advances on Bacterial Cellulose-Based Antibacterial Materials as Wound Dressings. Front. Bioeng. Biotechnol..

[98] Horue M., Silva J.M., Berti I.R., Brandão L.R., Barud H.d.S., Castro G.R. (2023). Bacterial Cellulose-Based Materials as Dressings for Wound Healing. Pharmaceutics.

[99] Czaja W.K., Young D.J., Kawecki M., Brown R.M. (2007). The Future Prospects of Microbial Cellulose in Biomedical Applications. Biomacromolecules.

[100] Ullah H., Wahid F., Santos H.A., Khan T. (2016). Advances in Biomedical and Pharmaceutical Applications of Functional Bacterial Cellulose-Based Nanocomposites. Carbohydr. Polym..

[101] Zhou C., Yang Z., Xun X., Ma L., Chen Z., Hu X., Wu X., Wan Y., Ao H. (2022). De Novo Strategy with Engineering a Multifunctional Bacterial Cellulose-Based Dressing for Rapid Healing of Infected Wounds. Bioact. Mater..

[102] Pasaribu K.M., Ilyas S., Tamrin T., Radecka I., Swingler S., Gupta A., Stamboulis A.G., Gea S. (2023). Bioactive Bacterial Cellulose Wound Dressings for Burns with Collagen In-Situ and Chitosan Ex-Situ Impregnation. Int. J. Biol. Macromol..

[103] Yuan H., Chen L., Hong F.F. (2020). A Biodegradable Antibacterial Nanocomposite Based on Oxidized Bacterial Nanocellulose for Rapid Hemostasis and Wound Healing. ACS Appl. Mater. Interfaces.

[104] Chiaoprakobkij N., Suwanmajo T., Sanchavanakit N., Phisalaphong M. (2020). Curcumin-Loaded Bacterial Cellulose/Alginate/Gelatin as A Multifunctional Biopolymer Composite Film. Molecules.

[105] Lamboni L., Li Y., Liu J., Yang G. (2016). Silk Sericin-Functionalized Bacterial Cellulose as a Potential Wound-Healing Biomaterial. Biomacromolecules.

[106] Aditya T., Allain J.P., Jaramillo C., Restrepo A.M. (2022). Surface Modification of Bacterial Cellulose for Biomedical Applications. Int. J. Mol. Sci..

[107] Radu C.D., Verestiuc L., Ulea E., Lipsa F.D., Vulpe V., Munteanu C., Bulgariu L., Pașca S., Tamas C., Ciuntu B.M. (2021). Evaluation of Keratin/Bacterial Cellulose Based Scaffolds as Potential Burned Wound Dressing. Appl. Sci..

[108] Sampaio L.M.P., Padrão J., Faria J., Silva J.P., Silva C.J., Dourado F., Zille A. (2016). Laccase Immobilization on Bacterial Nanocellulose Membranes: Antimicrobial, Kinetic and Stability Properties. Carbohydr. Polym..

[109] Kim S., Ko J., Choi J.H., Kang J.Y., Lim C., Shin M., Lee D.W., Kim J.W. (2022). Antigen–Antibody Interaction-Derived Bioadhesion of Bacterial Cellulose Nanofibers to Promote Topical Wound Healing. Adv. Funct. Mater..

[110] Vasconcelos N.F., Cunha A.P., Ricardo N.M.P.S., Freire R.S., Vieira L.d.A.P., Brígida A.I.S., Borges M.d.F., Rosa M.d.F., Vieira R.S., Andrade F.K. (2020). Papain Immobilization on Heterofunctional Membrane Bacterial Cellulose as a Potential Strategy for the Debridement of Skin Wounds. Int. J. Biol. Macromol..

[111] Bayazidi P., Almasi H., Asl A.K. (2018). Immobilization of Lysozyme on Bacterial Cellulose Nanofibers: Characteristics, Antimicrobial Activity and Morphological Properties. Int. J. Biol. Macromol..

[112] Costa de Oliveira Souza C.M., de Souza C.F., Mogharbel B.F., Irioda A.C., Cavichiolo Franco C.R., Sierakowski M.R., Athayde Teixeira de Carvalho K. (2021). Nanostructured Cellulose–Gellan–Xyloglucan–Lysozyme Dressing Seeded with Mesenchymal Stem Cells for Deep Second-Degree Burn Treatment. Int. J. Nanomed..

[113] dos Santos C.A., dos Santos G.R., Soeiro V.S., dos Santos J.R., Rebelo M.d.A., Chaud M.V., Gerenutti M., Grotto D., Pandit R., Rai M. (2018). Bacterial Nanocellulose Membranes Combined with Nisin: A Strategy to Prevent Microbial Growth. Cellulose.

[114] Rollini M., Musatti A., Cavicchioli D., Bussini D., Farris S., Rovera C., Romano D., De Benedetti S., Barbiroli A. (2020). From Cheese Whey Permeate to Sakacin-A/Bacterial Cellulose Nanocrystal Conjugates for Antimicrobial Food Packaging Applications: A Circular Economy Case Study. Sci. Rep..

[115] Malheiros P.S., Jozala A.F., Pessoa A., Vila M.M.D.C., Balcão V.M., Franco B.D.G.M. (2018). Immobilization of Antimicrobial Peptides from Lactobacillus Sakei Subsp. Sakei 2a in Bacterial Cellulose: Structural and Functional Stabilization. Food Packag. Shelf Life.

[116] Benevenuto L.G.D., da Silva Barud H., Cruz S.A., Caillier B., da Silva Paiva R., Achcar J.A., Montrezor L.H. (2023). Bacterial Cellulose-Based Cell Culture Platform Modified by Oxygen Plasma for Tissue Engineering Applications. Cellulose.

[117] Huang Y., Wang J., Yang F., Shao Y., Zhang X., Dai K. (2017). Modification and Evaluation of Micro-Nano Structured Porous Bacterial Cellulose Scaffold for Bone Tissue Engineering. Mater. Sci. Eng. C.

[118] Khan S., Ul-Islam M., Ikram M., Ullah M.W., Israr M., Subhan F., Kim Y., Jang J.H., Yoon S., Park J.K. (2016). Three-Dimensionally Microporous and Highly Biocompatible Bacterial Cellulose–Gelatin Composite Scaffolds for Tissue Engineering Applications. RSC Adv..

[119] Nagaoka M., Jiang H.-L., Hoshiba T., Akaike T., Cho C.-S. (2010). Application of Recombinant Fusion Proteins for Tissue Engineering. Ann. Biomed. Eng..

[120] Lee S.-H., An S.-J., Lim Y.-M., Huh J.-B. (2017). The Efficacy of Electron Beam Irradiated Bacterial Cellulose Membranes as Compared with Collagen Membranes on Guided Bone Regeneration in Peri-Implant Bone Defects. Materials.

[121] Xiong G., Luo H., Zhu Y., Raman S., Wan Y. (2014). Creation of Macropores in Three-Dimensional Bacterial Cellulose Scaffold for Potential Cancer Cell Culture. Carbohydr. Polym..

[122] Picheth G.F., Pirich C.L., Sierakowski M.R., Woehl M.A., Sakakibara C.N., de Souza C.F., Martin A.A., da Silva R., de Freitas R.A. (2017). Bacterial Cellulose in Biomedical Applications: A Review. Int. J. Biol. Macromol..

[123] Yadav V., Sun L., Panilaitis B., Kaplan D.L. (2015). In Vitro Chondrogenesis with Lysozyme Susceptible Bacterial Cellulose as a Scaffold. J. Tissue Eng. Regen. Med..

[124] Girard V., Chaussé J., Vermette P. (2024). Bacterial Cellulose: A Comprehensive Review. J. Appl. Polym. Sci..

[125] Osorio M., Cañas A., Puerta J., Díaz L., Naranjo T., Ortiz I., Castro C. (2019). Ex Vivo and In Vivo Biocompatibility Assessment (Blood and Tissue) of Three-Dimensional Bacterial Nanocellulose Biomaterials for Soft Tissue Implants. Sci. Rep..

[126] Acasigua G., Olyveira G., Costa L., Braghirolli D., Fossati A., Guastaldi A., Pranke P., Daltro G., Basmaji P. (2014). Novel Chemically Modified Bacterial Cellulose Nanocomposite as Potential Biomaterial for Stem Cell Therapy Applications. Curr. Stem Cell Res. Ther..

[127] Fu L., Zhou P., Zhang S., Yang G. (2013). Evaluation of Bacterial Nanocellulose-Based Uniform Wound Dressing for Large Area Skin Transplantation. Mater. Sci. Eng. C.

[128] Khan S., Ul-Islam M., Ullah M.W., Zhu Y., Narayanan K.B., Han S.S., Park J.K. (2022). Fabrication Strategies and Biomedical Applications of Three-Dimensional Bacterial Cellulose-Based Scaffolds: A Review. Int. J. Biol. Macromol..

[129] Pértile R.A.N., Moreira S., Gil da Costa R.M., Correia A., Guãrdao L., Gartner F., Vilanova M., Gama M. (2012). Bacterial Cellulose: Long-Term Biocompatibility Studies. J. Biomater. Sci. Polym. Ed..

[130] Castner D.G. (2017). Biomedical Surface Analysis: Evolution and Future Directions (Review). Biointerphases.

[131] Dugan J.M., Gough J.E., Eichhorn S.J. (2013). Bacterial Cellulose Scaffolds and Cellulose Nanowhiskers for Tissue Engineering. Nanomedicine.

[132] Zhijiang C., Guang Y. (2011). Bacterial Cellulose/Collagen Composite: Characterization and First Evaluation of Cytocompatibility. J. Appl. Polym. Sci..

[133] Albu M., Vuluga Z., Panaitescu D., Vuluga D., Căşărică A., Ghiurea M. (2014). Morphology and Thermal Stability of Bacterial Cellulose/Collagen Composites. Open Chem..

[134] Adhikari J., Dasgupta S., Barui A., Ghosh M., Saha P. (2023). Collagen Incorporated Functionalized Bacterial Cellulose Composite: A Macromolecular Approach for Successful Tissue Engineering Applications. Cellulose.

[135] Noh Y.K., Dos Santos Da Costa A., Park Y.S., Du P., Kim I.-H., Park K. (2019). Fabrication of Bacterial Cellulose-Collagen Composite Scaffolds and Their Osteogenic Effect on Human Mesenchymal Stem Cells. Carbohydr. Polym..

[136] Saska S., Teixeira L.N., Tambasco de Oliveira P., Minarelli Gaspar A.M., Lima Ribeiro S.J., Messaddeq Y., Marchetto R. (2012). Bacterial Cellulose-Collagen Nanocomposite for Bone Tissue Engineering. J. Mater. Chem..

[137] Svensson A., Nicklasson E., Harrah T., Panilaitis B., Kaplan D.L., Brittberg M., Gatenholm P. (2005). Bacterial Cellulose as a Potential Scaffold for Tissue Engineering of Cartilage. Biomaterials.

[138] Koike T., Sha J., Bai Y., Matsuda Y., Hideshima K., Yamada T., Kanno T. (2019). Efficacy of Bacterial Cellulose as a Carrier of BMP-2 for Bone Regeneration in a Rabbit Frontal Sinus Model. Materials.

[139] Gorgieva S., Hribernik S. (2019). Microstructured and Degradable Bacterial Cellulose–Gelatin Composite Membranes: Mineralization Aspects and Biomedical Relevance. Nanomaterials.

[140] Cakmak A.M., Unal S., Sahin A., Oktar F.N., Sengor M., Ekren N., Gunduz O., Kalaskar D.M. (2020). 3D Printed Polycaprolactone/Gelatin/Bacterial Cellulose/Hydroxyapatite Composite Scaffold for Bone Tissue Engineering. Polymers.

[141] Keskin Z., Sendemir Urkmez A., Hames E.E. (2017). Novel Keratin Modified Bacterial Cellulose Nanocomposite Production and Characterization for Skin Tissue Engineering. Mater. Sci. Eng. C.

[142] Pértile R., Moreira S., Andrade F., Domingues L., Gama M. (2012). Bacterial Cellulose Modified Using Recombinant Proteins to Improve Neuronal and Mesenchymal Cell Adhesion. Biotechnol. Prog..

[143] Yang J., Zhu Z., Liu Y., Zheng Y., Xie Y., Lin J., Cai T. (2022). Double-Modified Bacterial Cellulose/Soy Protein Isolate Composites by Laser Hole Forming and Selective Oxidation Used for Urethral Repair. Biomacromolecules.

[144] Zang S., Zhang R., Chen H., Lu Y., Zhou J., Chang X., Qiu G., Wu Z., Yang G. (2015). Investigation on Artificial Blood Vessels Prepared from Bacterial Cellulose. Mater. Sci. Eng. C.

[145] Fink H., Faxälv L., Molnár G.F., Drotz K., Risberg B., Lindahl T.L., Sellborn A. (2010). Real-Time Measurements of Coagulation on Bacterial Cellulose and Conventional Vascular Graft Materials. Acta Biomater..

[146] Roberts E.L., Abdollahi S., Oustadi F., Stephens E.D., Badv M. (2023). Bacterial-Nanocellulose-Based Biointerfaces and Biomimetic Constructs for Blood-Contacting Medical Applications. ACS Mater. Au.

[147] Klemm D., Schumann D., Udhardt U., Marsch S. (2001). Bacterial Synthesized Cellulose—Artificial Blood Vessels for Microsurgery. Prog. Polym. Sci..

[148] Lin N., Dufresne A. (2014). Nanocellulose in Biomedicine: Current Status and Future Prospect. Eur. Polym. J..

[149] Klemm D., Heublein B., Fink H., Bohn A. (2005). Cellulose: Fascinating Biopolymer and Sustainable Raw Material. Angew. Chem. Int. Ed..

[150] Brown E.E., Zhang J., Laborie M.-P.G. (2011). Never-Dried Bacterial Cellulose/Fibrin Composites: Preparation, Morphology and Mechanical Properties. Cellulose.

[151] Andrade F.K., Silva J.P., Carvalho M., Castanheira E.M.S., Soares R., Gama M. (2011). Studies on the Hemocompatibility of Bacterial Cellulose. J. Biomed. Mater. Res. Part A.

[152] Tibbitt M.W., Anseth K.S. (2009). Hydrogels as Extracellular Matrix Mimics for 3D Cell Culture. Biotechnol. Bioeng..

[153] Leitão A.F., Faria M.A., Faustino A.M.R., Moreira R., Mela P., Loureiro L., Silva I., Gama M. (2016). A Novel Small-Caliber Bacterial Cellulose Vascular Prosthesis: Production, Characterization, and Preliminary In Vivo Testing. Macromol. Biosci..

[154] Chaicharoenaudomrung N., Kunhorm P., Noisa P. (2019). Three-Dimensional Cell Culture Systems as an *in Vitro* Platform for Cancer and Stem Cell Modeling. World J. Stem Cells.

[155] Unal S., Arslan S., Yilmaz B.K., Oktar F.N., Sengil A.Z., Gunduz O. (2021). Production and Characterization of Bacterial Cellulose Scaffold and Its Modification with Hyaluronic Acid and Gelatin for Glioblastoma Cell Culture. Cellulose.

[156] Opwis K., Gutmann J.S. (2011). Surface Modification of Textile Materials with Hydrophobins. Text. Res. J..

[157] Wan Z., Wang L., Ma L., Sun Y., Yang X. (2017). Controlled Hydrophobic Biosurface of Bacterial Cellulose Nanofibers through Self-Assembly of Natural Zein Protein. ACS Biomater. Sci. Eng..

[158] Rijal G., Li W. (2016). 3D Scaffolds in Breast Cancer Research. Biomaterials.

[159] Reis E.M.d., Berti F.V., Colla G., Porto L.M. (2018). Bacterial Nanocellulose-IKVAV Hydrogel Matrix Modulates Melanoma Tumor Cell Adhesion and Proliferation and Induces Vasculogenic Mimicry in Vitro. J. Biomed. Mater. Res. Part B Appl. Biomater..

[160] Xiong G., Luo H., Gu F., Zhang J., Hu D., Wan Y. (2013). A Novel in Vitro Three-Dimensional Macroporous Scaffolds from Bacterial Cellulose for Culture of Breast Cancer Cells. J. Biomater. Nanobiotechnol..

[161] Luo H., Gu P., Xiong G., Hu D. (2016). A Multichanneled Bacterial Cellulose Scaffold for 3D in Vitro Cancer Culture. Cellul. Chem. Technol..

[162] Islam S.U., Ul-Islam M., Ahsan H., Ahmed M.B., Shehzad A., Fatima A., Sonn J.K., Lee Y.S. (2021). Potential Applications of Bacterial Cellulose and Its Composites for Cancer Treatment. Int. J. Biol. Macromol..

[163] Gao C., Wan Y., Yang C., Dai K., Tang T., Luo H., Wang J. (2011). Preparation and Characterization of Bacterial Cellulose Sponge with Hierarchical Pore Structure as Tissue Engineering Scaffold. J. Porous Mater..

[164] Ul-Islam M., Subhan F., Islam S.U., Khan S., Shah N., Manan S., Ullah M.W., Yang G. (2019). Development of Three-Dimensional Bacterial Cellulose/Chitosan Scaffolds: Analysis of Cell-Scaffold Interaction for Potential Application in the Diagnosis of Ovarian Cancer. Int. J. Biol. Macromol..

[165] Chen Y., Zhou X., Lin Q., Jiang D. (2014). Bacterial Cellulose/Gelatin Composites: In Situ Preparation and Glutaraldehyde Treatment. Cellulose.

[166] Lopes T.D., Riegel-Vidotti I.C., Grein A., Tischer C.A., Faria-Tischer P.C. (2014). de S. Bacterial Cellulose and Hyaluronic Acid Hybrid Membranes: Production and Characterization. Int. J. Biol. Macromol..

[167] de Oliveira S.A., da Silva B.C., Riegel-Vidotti I.C., Urbano A., de Sousa Faria-Tischer P.C., Tischer C.A. (2017). Production and Characterization of Bacterial Cellulose Membranes with Hyaluronic Acid from Chicken Comb. Int. J. Biol. Macromol..

[168] Caro-Astorga J., Walker K.T., Herrera N., Lee K.-Y., Ellis T. (2021). Bacterial Cellulose Spheroids as Building Blocks for 3D and Patterned Living Materials and for Regeneration. Nat. Commun..

[169] Innala M., Riebe I., Kuzmenko V., Sundberg J., Gatenholm P., Hanse E., Johannesson S. (2014). 3D Culturing and Differentiation of SH-SY5Y Neuroblastoma Cells on Bacterial Nanocellulose Scaffolds. Artif. Cells Nanomed. Biotechnol..

[170] Wang B., Ji P., Ma Y., Song J., You Z., Chen S. (2021). Bacterial Cellulose Nanofiber Reinforced Poly(Glycerol-Sebacate) Biomimetic Matrix for 3D Cell Culture. Cellulose.

[171] Wang J., Zhao L., Zhang A., Huang Y., Tavakoli J., Tang Y. (2018). Novel Bacterial Cellulose/Gelatin Hydrogels as 3D Scaffolds for Tumor Cell Culture. Polymers.

[172] Robbins M., Pisupati V., Azzarelli R., Nehme S.I., Barker R.A., Fruk L., Schierle G.S.K. (2021). Biofunctionalised Bacterial Cellulose Scaffold Supports the Patterning and Expansion of Human Embryonic Stem Cell-Derived Dopaminergic Progenitor Cells. Stem Cell Res. Ther..

[173] He M., Yan Y., Liu X., Li L., Yang B., Liu M., Yu Q., Wang E., Li P., Liu T. (2024). A Nanobody-Mediated Drug System against Largemouth Bass Virus Delivered by Bacterial Nanocellulose in Micropterus Salmoides. Int. J. Biol. Macromol..

[174] Liang S. (2023). Advances in Drug Delivery Applications of Modified Bacterial Cellulose-Based Materials. Front. Bioeng. Biotechnol..

[175] Zhang W., Wang X., Wang J., Zhang L. (2019). Drugs Adsorption and Release Behavior of Collagen/Bacterial Cellulose Porous Microspheres. Int. J. Biol. Macromol..

[176] Qi Z., Pei P., Zhang Y., Chen H., Yang S., Liu T., Zhang Y., Yang K. (2022). 131I-APD-L1 Immobilized by Bacterial Cellulose for Enhanced Radio-Immunotherapy of Cancer. J. Control. Release.

[177] Munawaroh H.S.H., Anwar B., Yuliani G., Murni I.C., Arindita N.P.Y., Maulidah G.S., Martha L., Hidayati N.A., Chew K.W., Show P.-L. (2023). Bacterial Cellulose Nanocrystal as Drug Delivery System for Overcoming the Biological Barrier of Cyano-Phycocyanin: A Biomedical Application of Microbial Product. Bioengineered.

[178] Estevinho B.N., Samaniego N., Talens-Perales D., Fabra M.J., López-Rubio A., Polaina J., Marín-Navarro J. (2018). Development of Enzymatically-Active Bacterial Cellulose Membranes through Stable Immobilization of an Engineered β-Galactosidase. Int. J. Biol. Macromol..

[179] Cacicedo M.L., Castro M.C., Servetas I., Bosnea L., Boura K., Tsafrakidou P., Dima A., Terpou A., Koutinas A., Castro G.R. (2016). Progress in Bacterial Cellulose Matrices for Biotechnological Applications. Bioresour. Technol..

[180] Fernandez-Lorente G., Lopez-Gallego F., Bolivar J.M., Rocha-Martin J., Moreno-Perez S., Guisan J.M. (2015). Immobilization of Proteins on Glyoxyl Activated Supports: Dramatic Stabilization of Enzymes by Multipoint Covalent Attachment on Pre-Existing Supports. Curr. Org. Chem..

[181] Drozd R., Szymańska M., Rakoczy R., Junka A., Szymczyk P., Fijałkowski K. (2019). Functionalized Magnetic Bacterial Cellulose Beads as Carrier for Lecitase® Ultra Immobilization. Appl. Biochem. Biotechnol..

[182] Brisola J., Andrade G.J.S., de Oliveira S.A., Viana R.M.R., Tischer P.C.d.S.F., Tischer C.A. (2022). Covalent Immobilization of Lipase on Bacterial Cellulose Membrane and Nanocellulose. Mater. Res..

[183] Kim H.J., Jin J.N., Kan E., Kim K.J., Lee S.H. (2017). Bacterial Cellulose-Chitosan Composite Hydrogel Beads for Enzyme Immobilization. Biotechnol. Bioprocess Eng..

[184] Cai Q., Hu C., Yang N., Wang Q., Wang J., Pan H., Hu Y., Ruan C. (2018). Enhanced Activity and Stability of Industrial Lipases Immobilized onto Spherelike Bacterial Cellulose. Int. J. Biol. Macromol..

[185] Yu B., Cheng H., Zhuang W., Zhu C., Wu J., Niu H., Liu D., Chen Y., Ying H. (2019). Stability and Repeatability Improvement of Horseradish Peroxidase by Immobilization on Amino-Functionalized Bacterial Cellulose. Process Biochem..

[186] Li G., Nandgaonkar A.G., Wang Q., Zhang J., Krause W.E., Wei Q., Lucia L.A. (2017). Laccase-Immobilized Bacterial Cellulose/TiO_2_ Functionalized Composite Membranes: Evaluation for Photo- and Bio-Catalytic Dye Degradation. J. Membr. Sci..

[187] Pesaran M., Amoabediny G., Yazdian F. (2015). Effect of Cultivation Time and Medium Condition in Production of Bacterial Cellulose Nanofiber for Urease Immobilization. Int. J. Polym. Sci..

[188] Shishparenok A.N., Koroleva S.A., Dobryakova N.V., Gladilina Y.A., Gromovykh T.I., Solopov A.B., Kudryashova E.V., Zhdanov D.D. (2024). Bacterial Cellulose Films for L-Asparaginase Delivery to Melanoma Cells. Int. J. Biol. Macromol..

[189] Gupta D.M.G. (2023). Polymer-Based Strategies for Enzyme Immobilization: A Comprehensive Review. Tuijin Jishu/J. Propuls. Technol..

[190] Akduman B., Uygun M., Çoban E.P., Uygun D.A., Bıyık H., Akgöl S. (2013). Reversible Immobilization of Urease by Using Bacterial Cellulose Nanofibers. Appl. Biochem. Biotechnol..

[191] Drozd R., Szymańska M., Przygrodzka K., Hoppe J., Leniec G., Kowalska U. (2021). The Simple Method of Preparation of Highly Carboxylated Bacterial Cellulose with Ni- and Mg-Ferrite-Based Versatile Magnetic Carrier for Enzyme Immobilization. Int. J. Mol. Sci..

[192] Pandey D., Daverey A., Arunachalam K. (2020). Biochar: Production, Properties and Emerging Role as a Support for Enzyme Immobilization. J. Clean. Prod..

[193] Overview of Crosslinking and Protein Modification. https://www.thermofisher.com/ru/ru/home/life-science/protein-biology/protein-biology-learning-center/protein-biology-resource-library/pierce-protein-methods/overview-crosslinking-protein-modification.html#3.

[194] Jackson E., Correa S., Betancor L. (2019). Cellulose-Based Nanosupports for Enzyme Immobilization. Cellulose-Based Superabsorbent Hydrogels. Polymers and Polymeric Composites: A Reference Series.

[195] Khozeymeh Nezhad M., Aghaei H. (2021). Tosylated Cloisite as a New Heterofunctional Carrier for Covalent Immobilization of Lipase and Its Utilization for Production of Biodiesel from Waste Frying Oil. Renew. Energy.

[196] Dutta K., Hu D., Zhao B., Ribbe A.E., Zhuang J., Thayumanavan S. (2017). Templated Self-Assembly of a Covalent Polymer Network for Intracellular Protein Delivery and Traceless Release. J. Am. Chem. Soc..

[197] Bora U., Kannan K., Nahar P. (2005). A Simple Method for Functionalization of Cellulose Membrane for Covalent Immobilization of Biomolecules. J. Membr. Sci..

[198] Schmidt M., Abdul Latif A., Prager A., Gläser R., Schulze A. (2022). Highly Efficient One-Step Protein Immobilization on Polymer Membranes Supported by Response Surface Methodology. Front. Chem..

[199] Birkheur S., Laureto E., Fernandes R.V., Tischer C., Butera A.P., Ribeiro-Viana R.M. (2020). Diphenyltetrazole Modified Bacterial Cellulose Film: Considerations on Heterogeneous Modification and Bioconjugation. Mater. Res..

[200] Vandamme E.J., De Baets S., Vanbaelen A., Joris K., De Wulf P. (1998). Improved Production of Bacterial Cellulose and Its Application Potential. Polym. Degrad. Stab..

[201] Chiaoprakobkij N., Seetabhawang S., Okhawilai M., Uyama H., Phisalaphong M. (2022). Multifunctional Bacterial Cellulose-Gelatin Containing Mangosteen Extract Films with Improved Antibacterial and Anticancer Properties. Cellulose.

[202] Stumpf T.R., Yang X., Zhang J., Cao X. (2018). In Situ and Ex Situ Modifications of Bacterial Cellulose for Applications in Tissue Engineering. Mater. Sci. Eng. C.

[203] Pang M., Huang Y., Meng F., Zhuang Y., Liu H., Du M., Ma Q., Wang Q., Chen Z., Chen L. (2020). Application of Bacterial Cellulose in Skin and Bone Tissue Engineering. Eur. Polym. J..

[204] Katepetch C., Rujiravanit R. (2011). Synthesis of Magnetic Nanoparticle into Bacterial Cellulose Matrix by Ammonia Gas-Enhancing in Situ Co-Precipitation Method. Carbohydr. Polym..

[205] Yano S., Maeda H., Nakajima M., Hagiwara T., Sawaguchi T. (2008). Preparation and Mechanical Properties of Bacterial Cellulose Nanocomposites Loaded with Silica Nanoparticles. Cellulose.

[206] Novogrodsky A. (1975). Induction of Lymphocyte Cytotoxicity by Modification of the Effector or Target Cells with Periodate or with Neuraminidase and Galactose Oxidase. J. Immunol..

[207] Badshah M., Ullah H., Khan A.R., Khan S., Park J.K., Khan T. (2018). Surface Modification and Evaluation of Bacterial Cellulose for Drug Delivery. Int. J. Biol. Macromol..

[208] Rouabhia M., Asselin J., Tazi N., Messaddeq Y., Levinson D., Zhang Z. (2014). Production of Biocompatible and Antimicrobial Bacterial Cellulose Polymers Functionalized by RGDC Grafting Groups and Gentamicin. ACS Appl. Mater. Interfaces.

[209] Lin Q., Zheng Y., Wang G., Shi X., Zhang T., Yu J., Sun J. (2015). Protein Adsorption Behaviors of Carboxymethylated Bacterial Cellulose Membranes. Int. J. Biol. Macromol..

[210] Oshima T., Kondo K., Ohto K., Inoue K., Baba Y. (2008). Preparation of Phosphorylated Bacterial Cellulose as an Adsorbent for Metal Ions. React. Funct. Polym..

[211] Azarniya A., Tamjid E., Eslahi N., Simchi A. (2019). Modification of Bacterial Cellulose/Keratin Nanofibrous Mats by a Tragacanth Gum-Conjugated Hydrogel for Wound Healing. Int. J. Biol. Macromol..

[212] Kim D.-Y., Nishiyama Y., Kuga S. (2002). Surface Acetylation of Bacterial Cellulose. Cellulose.

[213] Shen H., Liao S., Jiang C., Zhang J., Wei Q., Ghiladi R.A., Wang Q. (2022). In Situ Grown Bacterial Cellulose/MoS2 Composites for Multi-Contaminant Wastewater Treatment and Bacteria Inactivation. Carbohydr. Polym..

[214] Potočnik V., Gorgieva S., Trček J. (2023). From Nature to Lab: Sustainable Bacterial Cellulose Production and Modification with Synthetic Biology. Polymers.

[215] Serafica G., Mormino R., Bungay H. (2002). Inclusion of Solid Particles in Bacterial Cellulose. Appl. Microbiol. Biotechnol..

[216] Cheng K.-C., Catchmark J.M., Demirci A. (2009). Enhanced Production of Bacterial Cellulose by Using a Biofilm Reactor and Its Material Property Analysis. J. Biol. Eng..

[217] Ul-Islam M., Khan T., Park J.K. (2012). Water Holding and Release Properties of Bacterial Cellulose Obtained by in Situ and Ex Situ Modification. Carbohydr. Polym..

[218] Seifert M., Hesse S., Kabrelian V., Klemm D. (2004). Controlling the Water Content of Never Dried and Reswollen Bacterial Cellulose by the Addition of Water-soluble Polymers to the Culture Medium. J. Polym. Sci. Part A Polym. Chem..

[219] Gorgieva S. (2020). Bacterial Cellulose as a Versatile Platform for Research and Development of Biomedical Materials. Processes.

[220] Kim G.-D., Yang H., Park H.R., Park C.-S., Park Y.S., Lee S.E. (2013). Evaluation of Immunoreactivity of in Vitro and in Vivo Models against Bacterial Synthesized Cellulose to Be Used as a Prosthetic Biomaterial. BioChip J..

[221] Czaja W., Krystynowicz A., Bielecki S., Brownjr R. (2006). Microbial Cellulose—The Natural Power to Heal Wounds. Biomaterials.

[222] Fürsatz M., Skog M., Sivlér P., Palm E., Aronsson C., Skallberg A., Greczynski G., Khalaf H., Bengtsson T., Aili D. (2018). Functionalization of Bacterial Cellulose Wound Dressings with the Antimicrobial Peptide *ε*-Poly-L-Lysine. Biomed. Mater..

[223] Shah J.L., Li G., Shaffer J.L., Azoulay M.I., Gibbs I.C., Nagpal S., Soltys S.G. (2018). Stereotactic Radiosurgery and Hypofractionated Radiotherapy for Glioblastoma. Neurosurgery.

[224] Autier L., Clavreul A., Cacicedo M.L., Franconi F., Sindji L., Rousseau A., Perrot R., Montero-Menei C.N., Castro G.R., Menei P. (2019). A New Glioblastoma Cell Trap for Implantation after Surgical Resection. Acta Biomater..

[225] Andrade F.K., Morais J.P.S., Muniz C.R., Nascimento J.H.O., Vieira R.S., Gama F.M.P., Rosa M.F. (2019). Stable Microfluidized Bacterial Cellulose Suspension. Cellulose.

[226] Liyaskina E., Revin V., Paramonova E., Nazarkina M., Pestov N., Revina N., Kolesnikova S. (2017). Nanomaterials from Bacterial Cellulose for Antimicrobial Wound Dressing. J. Phys. Conf. Ser..

[227] Zhang X., Wang D., Liu S., Tang J. (2022). Bacterial Cellulose Nanofibril-Based Pickering Emulsions: Recent Trends and Applications in the Food Industry. Foods.

[228] Singhsa P., Narain R., Manuspiya H. (2018). Bacterial Cellulose Nanocrystals (BCNC) Preparation and Characterization from Three Bacterial Cellulose Sources and Development of Functionalized BCNCs as Nucleic Acid Delivery Systems. ACS Appl. Nano Mater..

[229] Li Q., Wang Y., Wu Y., He K., Li Y., Luo X., Li B., Wang C., Liu S. (2019). Flexible Cellulose Nanofibrils as Novel Pickering Stabilizers: The Emulsifying Property and Packing Behavior. Food Hydrocoll..

[230] Liu W., Du H., Zhang M., Liu K., Liu H., Xie H., Zhang X., Si C. (2020). Bacterial Cellulose-Based Composite Scaffolds for Biomedical Applications: A Review. ACS Sustain. Chem. Eng..

[231] Balea A., Fuente E., Monte M.C., Merayo N., Campano C., Negro C., Blanco A. (2020). Industrial Application of Nanocelluloses in Papermaking: A Review of Challenges, Technical Solutions, and Market Perspectives. Molecules.

[232] Rol F., Belgacem M.N., Gandini A., Bras J. (2019). Recent Advances in Surface-Modified Cellulose Nanofibrils. Prog. Polym. Sci..

[233] Li Q., Chen P., Li Y., Li B., Liu S. (2020). Construction of Cellulose-Based Pickering Stabilizer as a Novel Interfacial Antioxidant: A Bioinspired Oxygen Protection Strategy. Carbohydr. Polym..

[234] Vasconcelos N.F., Feitosa J.P.A., da Gama F.M.P., Morais J.P.S., Andrade F.K., de Souza Filho M.d.S.M., Rosa M.d.F. (2017). Bacterial Cellulose Nanocrystals Produced under Different Hydrolysis Conditions: Properties and Morphological Features. Carbohydr. Polym..

[235] Ribeiro-Viana R.M., Faria-Tischer P.C.S., Tischer C.A. (2016). Preparation of Succinylated Cellulose Membranes for Functionalization Purposes. Carbohydr. Polym..

[236] Oryan A., Kamali A., Moshiri A., Baharvand H., Daemi H. (2018). Chemical Crosslinking of Biopolymeric Scaffolds: Current Knowledge and Future Directions of Crosslinked Engineered Bone Scaffolds. Int. J. Biol. Macromol..

[237] Heck T., Faccio G., Richter M., Thöny-Meyer L. (2013). Enzyme-Catalyzed Protein Crosslinking. Appl. Microbiol. Biotechnol..

[238] Chanabodeechalermrung B., Chaiwarit T., Sommano S.R., Rachtanapun P., Kantrong N., Chittasupho C., Jantrawut P. (2022). Dual Crosslinked Ion-Based Bacterial Cellulose Composite Hydrogel Containing Polyhexamethylene Biguanide. Membranes.

[239] Shen X., Shamshina J.L., Berton P., Gurau G., Rogers R.D. (2016). Hydrogels Based on Cellulose and Chitin: Fabrication, Properties, and Applications. Green Chem..

[240] Wang J., Wan Y.Z., Luo H.L., Gao C., Huang Y. (2012). Immobilization of Gelatin on Bacterial Cellulose Nanofibers Surface via Crosslinking Technique. Mater. Sci. Eng. C.

[241] Castro C., Cordeiro N., Faria M., Zuluaga R., Putaux J.-L., Filpponen I., Velez L., Rojas O.J., Gañán P. (2015). In-Situ Glyoxalization during Biosynthesis of Bacterial Cellulose. Carbohydr. Polym..

[242] Chaiyasat A., Jearanai S., Moonmangmee S., Moonmangmee D., P Christopher L., Nur Alam M., Chaiyasat P. (2018). Novel Green Hydrogel Material Using Bacterial Cellulose. Orient. J. Chem..

[243] Meftahi A., Khajavi R., Rashidi A., Rahimi M.K., Bahador A. (2018). Preventing the Collapse of 3D Bacterial Cellulose Network via Citric Acid. J. Nanostruct. Chem..

[244] Sommer A., Dederko-Kantowicz P., Staroszczyk H., Sommer S., Michalec M. (2021). Enzymatic and Chemical Cross-Linking of Bacterial Cellulose/Fish Collagen Composites—A Comparative Study. Int. J. Mol. Sci..

[245] Hubbe M.A. (2019). Review of the Mechanistic Roles of Nanocellulose, Cellulosic Fibers, and Hydrophilic Cellulose Derivatives in Cellulose-Based Absorbents. Cellulose-Based Superabsorbent Hydrogels.

[246] Pal K., Paulson A.T., Rousseau D. (2013). Biopolymers in Controlled-Release Delivery Systems. Handbook of Biopolymers and Biodegradable Plastics.

[247] Bao L., Li C., Tang M., Chen L., Hong F.F. (2022). Potential of a Composite Conduit with Bacterial Nanocellulose and Fish Gelatin for Application as Small-Diameter Artificial Blood Vessel. Polymers.

[248] Delgado L.M., Bayon Y., Pandit A., Zeugolis D.I. (2015). To Cross-Link or Not to Cross-Link? Cross-Linking Associated Foreign Body Response of Collagen-Based Devices. Tissue Eng. Part B Rev..

[249] Treesuppharat W., Rojanapanthu P., Siangsanoh C., Manuspiya H., Ummartyotin S. (2017). Synthesis and Characterization of Bacterial Cellulose and Gelatin-Based Hydrogel Composites for Drug-Delivery Systems. Biotechnol. Rep..

[250] Brown E.E., Laborie M.-P.G., Zhang J. (2012). Glutaraldehyde Treatment of Bacterial Cellulose/Fibrin Composites: Impact on Morphology, Tensile and Viscoelastic Properties. Cellulose.

[251] Lees A., Nelson B.L., Mond J.J. (1996). Activation of Soluble Polysaccharides with 1-Cyano-4-Dimethylaminopyridinium Tetrafluoroborate for Use in Protein—Polysaccharide Conjugate Vaccines and Immunological Reagents. Vaccine.

[252] Mond J.J., Lees A., Snapper C.M. (1995). T Cell-Independent Antigens Type 2. Annu. Rev. Immunol..

[253] Shafer D.E., Toll B., Schuman R.F., Nelson B.L., Mond J.J., Lees A. (2000). Activation of Soluble Polysaccharides with 1-Cyano-4-Dimethylaminopyridinium Tetrafluoroborate (CDAP) for Use in Protein-Polysaccharide Conjugate Vaccines and Immunological Reagents. II. Selective Crosslinking of Proteins to CDAP-Activated Polysaccharides. Vaccine.

[254] Wacker M., Riedel J., Walles H., Scherner M., Awad G., Varghese S., Schürlein S., Garke B., Veluswamy P., Wippermann J. (2021). Comparative Evaluation on Impacts of Fibronectin, Heparin–Chitosan, and Albumin Coating of Bacterial Nanocellulose Small-Diameter Vascular Grafts on Endothelialization In Vitro. Nanomaterials.

[255] Akhtar M.F., Hanif M., Ranjha N.M. (2016). Methods of Synthesis of Hydrogels … A Review. Saudi Pharm. J..

[256] Mohite P.B., Adhav S.S. (2017). A Hydrogels: Methods of Preparation and Applications. Int. J. Adv. Pharm..

[257] Alven S., Aderibigbe B.A. (2020). Chitosan and Cellulose-Based Hydrogels for Wound Management. Int. J. Mol. Sci..

[258] Mohd Amin M.C.I., Ahmad N., Halib N., Ahmad I. (2012). Synthesis and Characterization of Thermo- and PH-Responsive Bacterial Cellulose/Acrylic Acid Hydrogels for Drug Delivery. Carbohydr. Polym..

[259] Jing W., Chunxi Y., Yizao W., Honglin L., Fang H., Kerong D., Yuan H. (2013). Laser Patterning of Bacterial Cellulose Hydrogel and Its Modification With Gelatin and Hydroxyapatite for Bone Tissue Engineering. Soft Mater..

[260] Mohamad N., Mohd Amin M.C.I., Pandey M., Ahmad N., Rajab N.F. (2014). Bacterial Cellulose/Acrylic Acid Hydrogel Synthesized via Electron Beam Irradiation: Accelerated Burn Wound Healing in an Animal Model. Carbohydr. Polym..

[261] Ahmad N., Amin M.C.I.M., Mahali S.M., Ismail I., Chuang V.T.G. (2014). Biocompatible and Mucoadhesive Bacterial Cellulose *-g-* Poly(Acrylic Acid) Hydrogels for Oral Protein Delivery. Mol. Pharm..

[262] Lai C., Zhang S., Chen X., Sheng L. (2014). Nanocomposite Films Based on TEMPO-Mediated Oxidized Bacterial Cellulose and Chitosan. Cellulose.

[263] Credou J., Berthelot T. (2014). Cellulose: From Biocompatible to Bioactive Material. J. Mater. Chem. B.

[264] Cui Q., Zheng Y., Lin Q., Song W., Qiao K., Liu S. (2014). Selective Oxidation of Bacterial Cellulose by NO_2_–HNO_3_. RSC Adv..

[265] Zheng Y., Wen X., Wu J., Wang L.-N., Yuan Z., Peng J., Meng H. (2015). Immobilization of Collagen Peptide on Dialdehyde Bacterial Cellulose Nanofibers via Covalent Bonds for Tissue Engineering and Regeneration. Int. J. Nanomed..

[266] Luz E.P.C.G., Chaves P.H.S., Vieira L.d.A.P., Ribeiro S.F., Borges M.d.F., Andrade F.K., Muniz C.R., Infantes-Molina A., Rodríguez-Castellón E., Rosa M.d.F. (2020). In Vitro Degradability and Bioactivity of Oxidized Bacterial Cellulose-Hydroxyapatite Composites. Carbohydr. Polym..

[267] Li J., Wan Y., Li L., Liang H., Wang J. (2009). Preparation and Characterization of 2,3-Dialdehyde Bacterial Cellulose for Potential Biodegradable Tissue Engineering Scaffolds. Mater. Sci. Eng. C.

[268] Zhang W., Wang X., Li X., Zhang L., Jiang F. (2020). A 3D Porous Microsphere with Multistage Structure and Component Based on Bacterial Cellulose and Collagen for Bone Tissue Engineering. Carbohydr. Polym..

[269] Zeng N., Chen H., Wu Y., Liu Z. (2022). Adipose Stem Cell-Based Treatments for Wound Healing. Front. Cell Dev. Biol..

[270] Bian D., Wu Y., Song G., Azizi R., Zamani A. (2022). The Application of Mesenchymal Stromal Cells (MSCs) and Their Derivative Exosome in Skin Wound Healing: A Comprehensive Review. Stem Cell Res. Ther..

[271] Zhu Z., Yang J., Ji X., Wang Z., Dai C., Li S., Li X., Xie Y., Zheng Y., Lin J. (2022). Clinical Application of a Double-Modified Sulfated Bacterial Cellulose Scaffold Material Loaded with FGFR2-Modified Adipose-Derived Stem Cells in Urethral Reconstruction. Stem Cell Res. Ther..

[272] Jia Y., Zhai X., Fu W., Liu Y., Li F., Zhong C. (2016). Surfactant-Free Emulsions Stabilized by Tempo-Oxidized Bacterial Cellulose. Carbohydr. Polym..

[273] do Nascimento E.S., Pereira A.L.S., Barros M.d.O., Barroso M.K.d.A., Lima H.L.S., Borges M.d.F., Feitosa J.P.d.A., de Azeredo H.M.C., Rosa M.d.F. (2019). TEMPO Oxidation and High-Speed Blending as a Combined Approach to Disassemble Bacterial Cellulose. Cellulose.

[274] Khattak S., Qin X.-T., Wahid F., Huang L.-H., Xie Y.-Y., Jia S.-R., Zhong C. (2021). Permeation of Silver Sulfadiazine Into TEMPO-Oxidized Bacterial Cellulose as an Antibacterial Agent. Front. Bioeng. Biotechnol..

[275] Koshy T.M., Gowda D.V., Tom S., Karunakar G., Srivastava A., Moin A. (2016). Polymer Grafting-An Overview. Am. J. PharmTech Res..

[276] Stepanova M., Korzhikova-Vlakh E. (2022). Modification of Cellulose Micro- and Nanomaterials to Improve Properties of Aliphatic Polyesters/Cellulose Composites: A Review. Polymers.

[277] Hamedi S., Shojaosadati S.A., Najafi V., Alizadeh V. (2020). A Novel Double-Network Antibacterial Hydrogel Based on Aminated Bacterial Cellulose and Schizophyllan. Carbohydr. Polym..

[278] Hettegger H., Sumerskii I., Sortino S., Potthast A., Rosenau T. (2015). Silane Meets Click Chemistry: Towards the Functionalization of Wet Bacterial Cellulose Sheets. ChemSusChem.

[279] Patoary M.K., Islam S.R., Farooq A., Rashid M.A., Sarker S., Hossain M.Y., Rakib M.A.N., Al-Amin M., Liu L. (2023). Phosphorylation of Nanocellulose: State of the Art and Prospects. Ind. Crops Prod..

[280] Oshima T., Taguchi S., Ohe K., Baba Y. (2011). Phosphorylated Bacterial Cellulose for Adsorption of Proteins. Carbohydr. Polym..

[281] Müller D., Mandelli J.S., Marins J.A., Soares B.G., Porto L.M., Rambo C.R., Barra G.M.O. (2012). Electrically Conducting Nanocomposites: Preparation and Properties of Polyaniline (PAni)-Coated Bacterial Cellulose Nanofibers (BC). Cellulose.

[282] Luo H., Xiong G., Huang Y., He F., Wang Y., Wan Y. (2008). Preparation and Characterization of a Novel COL/BC Composite for Potential Tissue Engineering Scaffolds. Mater. Chem. Phys..

[283] Gao G., Cao Y., Zhang Y., Wu M., Ma T., Li G. (2020). In Situ Production of Bacterial Cellulose/Xanthan Gum Nanocomposites with Enhanced Productivity and Properties Using Enterobacter Sp. FY-07. Carbohydr. Polym..

[284] Cheng K.-C., Catchmark J.M., Demirci A. (2009). Effect of Different Additives on Bacterial Cellulose Production by Acetobacter Xylinum and Analysis of Material Property. Cellulose.

[285] de Lima Fontes M., Meneguin A.B., Tercjak A., Gutierrez J., Cury B.S.F., dos Santos A.M., Ribeiro S.J.L., Barud H.S. (2018). Effect of in Situ Modification of Bacterial Cellulose with Carboxymethylcellulose on Its Nano/Microstructure and Methotrexate Release Properties. Carbohydr. Polym..

[286] Orelma H., Morales L.O., Johansson L.-S., Hoeger I.C., Filpponen I., Castro C., Rojas O.J., Laine J. (2014). Affibody Conjugation onto Bacterial Cellulose Tubes and Bioseparation of Human Serum Albumin. RSC Adv..

[287] Jiang Y., Yu G., Zhou Y., Liu Y., Feng Y., Li J. (2020). Effects of Sodium Alginate on Microstructural and Properties of Bacterial Cellulose Nanocrystal Stabilized Emulsions. Colloids Surf. A Physicochem. Eng. Asp..

[288] Zhou L.L., Sun D.P., Hu L.Y., Li Y.W., Yang J.Z. (2007). Effect of Addition of Sodium Alginate on Bacterial Cellulose Production by *Acetobacter xylinum*. J. Ind. Microbiol. Biotechnol..

[289] Gilmour K.A., Aljannat M., Markwell C., James P., Scott J., Jiang Y., Torun H., Dade-Robertson M., Zhang M. (2023). Biofilm Inspired Fabrication of Functional Bacterial Cellulose through Ex-Situ and in-Situ Approaches. Carbohydr. Polym..

[290] Morris R.J., Schor M., Gillespie R.M.C., Ferreira A.S., Baldauf L., Earl C., Ostrowski A., Hobley L., Bromley K.M., Sukhodub T. (2017). Natural Variations in the Biofilm-Associated Protein BslA from the Genus Bacillus. Sci. Rep..

[291] Pandey A., Singh M.K., Singh A. (2023). Bacterial Cellulose: A Smart Biomaterial for Biomedical Applications. J. Mater. Res..

[292] Shabanpour B., Kazemi M., Ojagh S.M., Pourashouri P. (2018). Bacterial Cellulose Nanofibers as Reinforce in Edible Fish Myofibrillar Protein Nanocomposite Films. Int. J. Biol. Macromol..

[293] Ullah H., Santos H.A., Khan T. (2016). Applications of Bacterial Cellulose in Food, Cosmetics and Drug Delivery. Cellulose.

[294] Müller A., Ni Z., Hessler N., Wesarg F., Müller F.A., Kralisch D., Fischer D. (2013). The Biopolymer Bacterial Nanocellulose as Drug Delivery System: Investigation of Drug Loading and Release Using the Model Protein Albumin. J. Pharm. Sci..

[295] Gorgieva S., Trček J. (2019). Bacterial Cellulose: Production, Modification and Perspectives in Biomedical Applications. Nanomaterials.

[296] Qiao N., Fan X., Hu S., Zhang X., Wang L., Du Y., Wang L., Zhang X., Yu D. (2021). Bacterial Cellulose as an Oleaginous Yeast Cell Carrier for Soybean Oil Refinery Effluent Treatment and Pyrolysis Oil Production. Bioprocess Biosyst. Eng..

[297] Islam M.U., Ullah M.W., Khan S., Shah N., Park J.K. (2017). Strategies for Cost-Effective and Enhanced Production of Bacterial Cellulose. Int. J. Biol. Macromol..

[298] Hussain Z., Sajjad W., Khan T., Wahid F. (2019). Production of Bacterial Cellulose from Industrial Wastes: A Review. Cellulose.

[299] Avcioglu N.H. (2022). Bacterial Cellulose: Recent Progress in Production and Industrial Applications. World J. Microbiol. Biotechnol..

[300] Aragão J.V.S., Costa A.F.S., Silva G.L., Silva S.M., Macêdo J.S., Galdino C.J.S., Milanez V.F.A., Sarubbo L.A. (2020). Analysis of the Environmental Life Cycle of Bacterial Cellulose Production. Ital. Assoc. Chem. Eng..

[301] Weiss M., Haufe J., Carus M., Brandão M., Bringezu S., Hermann B., Patel M.K. (2012). A Review of the Environmental Impacts of Biobased Materials. J. Ind. Ecol..

[302] Kamal T., Ul-Islam M., Fatima A., Ullah M.W., Manan S. (2022). Cost-Effective Synthesis of Bacterial Cellulose and Its Applications in the Food and Environmental Sectors. Gels.

[303] Moradi M., Jacek P., Farhangfar A., Guimarães J.T., Forough M. (2021). The Role of Genetic Manipulation and in Situ Modifications on Production of Bacterial Nanocellulose: A Review. Int. J. Biol. Macromol..

[304] Liu K., Catchmark J.M. (2019). Bacterial Cellulose/Hyaluronic Acid Nanocomposites Production through Co-Culturing Gluconacetobacter Hansenii and Lactococcus Lactis in a Two-Vessel Circulating System. Bioresour. Technol..

[305] Hu H., Catchmark J.M., Demirci A. (2021). Co-Culture Fermentation on the Production of Bacterial Cellulose Nanocomposite Produced by Komagataeibacter Hansenii. Carbohydr. Polym. Technol. Appl..

[306] Eslahi N., Mahmoodi A., Mahmoudi N., Zandi N., Simchi A. (2020). Processing and Properties of Nanofibrous Bacterial Cellulose-Containing Polymer Composites: A Review of Recent Advances for Biomedical Applications. Polym. Rev..

[307] Torres F., Commeaux S., Troncoso O. (2012). Biocompatibility of Bacterial Cellulose Based Biomaterials. J. Funct. Biomater..

[308] Zhong C. (2020). Industrial-Scale Production and Applications of Bacterial Cellulose. Front. Bioeng. Biotechnol..

