# Supplementary material for: Protein Immobilization on Bacterial Cellulose for Biomedical Application
Source: Polymers (Basel). 2024 Aug 30;16(17):2468. doi: 10.3390/polym16172468 (PMC11397966; doi:10.3390/polym16172468)
Supplement: Supplementary file 1 [file polymers-16-02468-s001.zip › polymers-3126385-supplementary.pdf]

Table S1. Summary of BC modifications for protein immobilization in biomedicine.

Application	Immobilized protein and other components	Type of modification BC	Type of immobilization of protein	Features improved	References
Wound healing	ECM (collagen, elastin, and hyaluronan) and growth factors (B-FGF, H-EGF, and KGF)	BC + alginate solution	Physical adsorption	The ECM and growth factors were well-integrated with BC and gradually released in a timely manner. Modified BC had biocompatibility with human fibroblast *in vitro*	[70]
	Soybean isolate protein	Native BC	Physical adsorption	The addition of soy protein to BC improved fibroblast adhesion and proliferation and type I collagen expression *in vitro* compared to the control. The composites also promoted accelerated wound healing, collagen deposition, and enhanced angiogenesis and regeneration of skin and hair follicles in rats *in vivo*	[13]
	Laccase	Native BC	Physical adsorption	Antibacterial properties of a composite for *S. aureus* (92%) and *E. coli* (26%)	[108]
	Silk Sericin	Native BC	Physical adsorption	The addition of sericin to BC had no effect on the mechanical properties of BC or the adhesion and proliferation of keratinocytes culture in vitro	[105]
	Involucrin antibody (SY5)	TEMPO oxidized BCNF	Covalent bond via EDC/ NHS coupling reaction	SY5-conjugated BCNF effectively adheres to the skin surface and promotes wound healing	[109]
Antimicrobial activity	Papain	Sodium periodate oxidized BC	Covalent bond via aldehyde group of BC + physical adsorption	The OBC carrier was able to immobilize papain with a recovered activity of 93.3%, an immobilization efficiency of 49.4%, and superior	[110]

				thermal properties over BC	
	Lysozyme	BCNF	Physical adsorption	Improved storage stability, activity was retained more than 70% of its original activity after 9 cycles of use. Enhanced antimicrobial activity against *S. aureus, E. coli, L. monocytogenes, Y. entrocolitica, A. niger, S. sereviseae*	[111]
	Nisin	Native BC	Physical adsorption	Nisin in combination with EDTA exhibited significant antimicrobial and antioxidant activity against *S. aureus* and *E. coli*	[113]
	Sakacyn A	BCNC	Physical adsorption	The antimicrobial BCNC-Sakacyn A composite was found to be effective in reducing the Listeria population in storage trials carried out on a fresh Italian soft cheese	[114]
Tissue engineerin g	Gelatin	Native BC was dissolved in NMMO, after which the resulting solution was added porogen and gelatin	Mixing	The resulting composite had high porosity, rapid swelling and cell penetration. Prolonged cell incubation in 3D BC–gelatin scaffolds led to the production of ECM	[118]
	Zein	BCNF	Physical adsorption with followed evaporation-induced self-assembly (EISA) of zein molecules at 25 °C and hot-pressing	The addition of zein at low concentrations (5 mg/mL) resulted in an increase in surface roughness and high hydrophobicity of BCNF and did not result in a significant change in the internal structure and mechanical properties of BCNF. In comparison with pure BC, the BCNF-zein composites exhibited a significantly	[157]

				increased adhesion and proliferation of fibroblast cells.	
Skin tissue engineering	Keratin	Native BC (for ex situ modification)	Physical adsorption for *ex situ* modification or *in situ* BC/keratin production	*In vitro* experiments have shown that fibroblast cells and keratinocytes have significantly higher adhesion and proliferation in the presence of keratin on post-modified BC/keratin nanocomposites. Keratinocytes also retain their native epithelial morphology	[141]
Tissue regeneration	Gelatin	Sodium periodate oxidized BC	Physical adsorption	BC-gelatin membranes had high swelling, degradation rate, and pH retention. MRC-5 cells adhered to BC-gelatin membranes well and these membranes did not decrease the dehydrogenase activity cells	[139]
Bone tissue regeneration	Gelatin	Native BC	Physical adsorption or procyanidine crosslinking for the preparation of a BC/procyanidine/gelatin/ hydroxyapatite scaffold	Improvement of mechanical properties was observed in BC/procyanidine/gelatin/ hydroxyapatite scaffolds. This scaffold exhibited superior adhesion, viability, proliferation, and osteogenic differentiation of human bone marrow stromal cells. In vivo studies demonstrated that BC/procyanidine/gelatin/ hydroxyapatite composite exhibited the most favorable osteogenic properties	[117]
	Gelatin polycaprolactone (PCL), hydroxyapatite (HA)	BCNC	Components were physically mixed	The 3D-printed scaffolds with an 80% infill rate exhibited a pore size (~300 μm) suitable for bone tissue engineering. The incorporation of BC and HA into the PCL/GEL scaffold enhanced cell	[140]

				proliferation and attachment	
Bone tissue regeneration	Osteopontin	RAFT polymerization	Covalent bond via surface grafting of PAA	Enhancement of osteogenic differentiation of human periodontal ligament stem cells without an adverse effect on their morphology	[15]
Bone tissue engineering	BMP-2	Native BC	Physical adsorption	BC maintained the graft space and released BMP-2 in a sustained manner. A significantly greater newly generated bone was observed in the rabbits in the BC+BMP-2 group than in the rabbits in the BC and BMP-2 groups. There was no observed inflammation and no capsule development around the BC and BC-BMP-2	[138]
Bone tissue engineering	BMP-2	Sodium periodate oxidized homogenized BC. BC/collagen composite was prepared by a Schiff-base reaction	Physical adsorption	These COL/BC/BMP-2 microspheres showed biocompatibility and enhanced the adhesion, proliferation, and differentiation of the mouse osteogenic cell line MC3T3-E1 cells	[268]
Nerve tissue engineering	The recombinant protein (19)IKVAV-CBM3 and IKVAV-CBM3 proteins	Native BC	Physical adsorption	The recombinant protein (19)IKVAV-CBM3 improved the adherence for PC12 cells and 30% for mesenchymal stem cells. (19)IKVAV-CBM3 enabled the release of MSC-secreted NGF into the culture medium	[142]
Urethral scaffold	Soybean protein isolate (SPI)	Physical modification with laser and subsequent oxidation with	Covalent bond with BC	The results demonstrated that DMBC/SPI composite was effectively biodegradable, did not elicit a mild inflammatory response, and exhibited excellent compatibility with rabbit	[143]

		sodium periodate		urethra. The repaired urethra showed a smooth and continuous appearance	
Urethral reconstruction	Overexpressed $FGFR_2$-adipose-derived stem cells	Sodium periodate oxidized BC	-	$FGFR_2$ enhanced the osteogenic capacity of adipose-derived stem cells without greatly affecting lipogenic capacity	[271]
Hemocompatible material	Tripeptide Arg-Gly-Asp (RGD)	Native BC	Physical adsorption	The presence of RGD on the BC polymer improved platelet adherence. However, when endothelial cells were grown over RGD-treated BC, a confluent cell layer developed and nearly no platelets stuck to the surface	[151]
Artificial blood vessels	Fibrin	BC/fibrin composites were treated with GA	Covalent bond with BC	The strain at break of the GA-treated BC/fibrin composite was significantly lower than that of the native blood vessel, but the tensile stress-strain curve of the composite material exhibited an initial low modulus plateau, which was also demonstrated in the native blood vessel. The time-dependent viscoelastic behavior and reducing modulus of composites were comparable to those of small-diameter blood vessels.	[250]
	Gelatin	BC/gelatine composites were treated with GA	Covalent bond with BC	The BC/gelatin composite tubes could withstand pressure three times or higher than the human normal blood pressure. The axial tensile strength and elongation at break for BC/gelatin composite tubes were superior to that of the BC tube. Whole blood coagulation tests demonstrated that BC/gelatin tubes had a hemolysis rate of less	[247]

				than 1.0%, meeting the requirements for implantable biomedical devices	
Acellular tissue-engineered vascular grafts	Albumin or fibronectin	BC surface was activated with CDAP	Isourea bonds to the amino acids of the protein	Fibronectin coating significantly promoted the adhesion and growth of VECs and EPCs, while albumin only promoted the adhesion of VECs, but the cells were functionally impaired. At the same time, fibronectin-modified surfaces were capable of capturing platelets, which likely leads to increased thrombogenicity	[254]
Scaffolds for 3D in vitro culture	Gelatin	In situ modification of BC with hyaluronic acid and gelatin	Gelatin added to cultivation medium of *G. xylinus*	The BC/HA/Gel composite scaffolds showed that U251 glioblastoma cells had normal morphology and could grow, proliferate, and adhere to the scaffolds. Moreover, such cells in BC/HA/Gel scaffolds showed superior vitality and formed multilayered growth and compacted cell clusters	[155]
	BDNF, GDNF, laminin	Amino-functionalized BC using GPTMS	Covalent bond	The covalent functionalization of BC with growth factors facilitated the development and differentiation of human pluripotent stem cells into VM progenitor cells.	[172]
Cancer cell entrapment	HSA	Native BC	Physical adsorption	The BC membrane was capable of trapping F98 tumor cells, preventing their migration. F98 cells that were trapped on BC remained viable and retained the ability to grow	[224]
Controlled drug delivery	IgG antibodies, anti-	Native BC	Injection of antibodies	IgG was released within 24–48 hours *in vitro*. BC did not have a cytotoxic effect on M38 cell line	[32]

	CTLA-4 antibodies			and did not cause activation of dendritic cells. BC hydrogels significantly reduced the levels of IgG and anti-CTLA-4 antibodies *in vivo*. The antibodies immobilized on the BC retained their binding capacity after 14 days of implantation	
	131I-αPD-L1 antibody	BCNF	Physical adsorption	It was confirmed the distal tumor growth inhibition effect in the subcutaneous models. Long-term treatment of primary tumors with 131I-αPD-L1/BC, T cells in lymph nodes were polarized to CD8+ CTL. These cells then killed cancer cells and prevented cancer from spreading in the model	[176]
Targeted drug system against virus	Nanobody NbE4 and ribavirin	TEMPO oxidation	Covalent bond via EDC/ NHS coupling reaction	The results of RT-qPCR demonstrated that NbE4 markedly augmented the antiviral efficacy of ribavirin in both *in vitro* and *in vivo*. The targeted drug delivery system BC-Ribavirin-NbE4 mitigated the inflammatory response associated with LMBV infection and elevated the survival rate in comparison to other experimental groups	[173]
Drug delivery system	Cyano-phycocyanin	BCNC crosslinked by GA	Physical adsorption	Optimum adsorption on crosslinked BCNC reached 65.3% in 3 h. Crosslinked BCNC had larger pores than the original BCNC along with higher thermal stability and improved the long-time stability, resulting in slower drug release	[177]
	L-asparaginase	Native BC	Physical adsorption	The adsorption of L-asparaginase on BC films was 84.5 ± 5.7%. L-	[188]

				asparaginase immobilized on BC caused the death of more than 90% of uveal melanoma cells (A875) after 72 hours	
Oral delivery of proteins	BSA	BC-g-poly(acrylic acid) hydrogels were synthesized by electron beam irradiation	Physical adsorption	The cumulative release of BSA was less than 10% in simulated gastric fluid, demonstrating the ability of the hydrogels to protect BSA from the acidic environment of the stomach. An *ex vivo* penetration study showed that BSA penetration increased throughout the intestinal mucosal tissue. The hydrogels had no acute oral toxicity *in vivo*	[261]
Enzyme immobilization	Lipase	Native BC or BCNC functionalized with succinic acid as linker	Covalent bond via EDC/NHS coupling reaction	The immobilized enzyme retained its activity in both BCNC and BC membrane, and the amount of protein immobilized on BCNC was 2.75 times higher than that in BC membrane	[182]
	Hybrid β-galactosidase with CBM2 module	Native BC (hydrated or dried)	Affinity bond via CBM2 module	The immobilized hybrid β-galactosidase had a higher affinity and high specificity compared to the wild-type enzyme; efficiency of lactose hydrolysis was similar. The reuse of the hybrid enzyme was constrained by the instability of the β-galactosidase module, whereas the attachment of CBM2 to cellulose was stable even at high temperatures	[178]
	HRP	Amino-functionalized BC using APTES	GA-based crosslinking adsorption	Stability and repeatability improvement of HRP. The optimum pH range for immobilized HRP was wider than that of free enzyme	[185]

	Lipase	Sodium periodate -oxidized spherical BC	Physical adsorption and covalent binding	The stability, hydrolytic activity and active temperature of lipase immobilized by covalent binding on spherical BC were significantly improved	[184]
	SOD	Native BC	Physical adsorption	The SOD immobilization on BC increased its stability at high temperatures and protect in fibroblasts against oxidative damage	[4]
	Laccase and TiO_2	Sodium periodate -oxidized BC	Covalent bond via aldehyde group of BC	The immobilized laccase demonstrated superior pH and temperature stability compared to the free laccase. Furthermore, the immobilized laccase exhibited a relative activity of 67% after 10 cycles. In UV irradiation, the oxidized BC/TiO2-laccase composite degraded 95% of the dye within 3 hours	[186]
	GAD	Preactivation GA followed by adsorption	Physical adsorption or preactivation followed by adsorption, or adsorption followed by crosslinking	In the case of preactivation GA followed by adsorption, the immobilized enzyme retained more than 96% of its original activity after seven cycles and exhibited 89.17% of the activity of the native enzyme. Adsorption and crosslinking resulted in a GAD activity on BC of 60.1% of its starting value, which declined progressively during seven cycles. The physical adsorption approach demonstrated the highest GAD activity (95.11%), yet the enzyme activity after three cycles of usage was only 5% of the entire activity	[45]
	Lipase (*Candida rugosa)*	BCNF-chitosan hydrogel	Physical adsorption and crosslinking	The cross-linked lipases showed higher stability than adsorbed or free lipases. The highest	[183]

		beads (BC2)		thermal stability was observed for the lipase cross-linked to BC2. The residual activity of BC2 was 44% after incubation for 10 h, while that of free lipase was 15%. Half-life of lipase cross-linked to BC-chitosan beads at 60°C was 22.7 times that of free lipase	
	Lipase	BC activation by GA	Physical adsorption	The activity of the immobilized enzyme was 93.5% of that of the free enzyme. After six reactions, the activity was 76.7% (330 U) of that in the first reaction. The immobilized lipase retained 60% of its initial activity after 15 cycles of use, in contrast to the free enzyme immobilized and free enzymes had identical activity at different pH and temperature levels.	[14]
	Lecitase® Ultra enzyme	Sodium periodate -oxidized BC was saturated with PEI and a mixture of Fe^{2+}/Fe^{3+} ions. Then OBC was activated with GA	Covalent bond with GA-activated BC	The immobilization technique had no significant impact on the enzyme's K_M value, and the immobilized enzyme retained more than 70% of its original activity after 8 cycles of use. The immobilized enzyme demonstrated excellent storage stability, retaining 80% of its initial activity after four weeks at 4 °C	[181]
	Urease	BCNC	Physical adsorption with subsequently the GA crosslinking	The immobilized urease demonstrated tolerance to fluctuations in pH and temperature, as well as enhanced reusability compared to the free enzyme	[187]
	Lipase B from	BCNFwas cross-	EDC-mediated covalent linkage to BC-CA-	Both enzymes demonstrated	[191]

	Candida antarctica and phospholipase A *from Aspergillus oryzae*	linked by CA in presence of sodium hypophosphate. $MgFe_2O_4$ and $NiFe_2O_4$ magnetic particles were introduced into BC-CA	$NiFe_2O_4$, and BC-CA-$MgFe_2O_4$ composites	significantly enhanced thermal stability at 60 °C, with a notable retention of residual activity at 70 °C, where a significant decline was observed for the free form. The immobilized enzymes exhibited a notable degree of residual activity after ten cycles of repeated use. However, phospholipase A and lipase had decreased catalytic efficiency	
Biofiltration	anti-HSA affibody	In situ modification of BC with CMC or TEMPO oxidation	Covalent bond via EDC/ NHS coupling reaction	Affibody conjugation increased the affinity and specificity of CMC-BC tubes to capture target molecules	[286]
Adsorbent	Lysozyme	Phosphorylated BC	Physical adsorption	The amount of adsorbed lysozyme increased as the percentage of phosphorylation of the PBC adsorbent increased. PBC had a greater adsorption capacity for lysozyme than phosphorylated plant cellulose due to its larger surface area.	[280]
